# A multi-composite disease activity score (*NFKB1*-DAS) for *NFKB1* insufficiency

**DOI:** 10.1007/s10875-026-02081-3

**Published:** 2026-10-19

**Authors:** Katharina Thoma, Pia Hassunah, Manfred Fliegauf, Gabriele Müller, Andres Caballero Garcia de Oteyza, Pavla Mrovecova, Helene Kraus, Prerana Agarwal, Maximilian Frederik Russe, Birgit Sawitzki, Lev Petrov, Katrin Vogt, Nadezhda Camacho Ordonez, Aleksandra Hirsch, Christoph B. Geier, Máté Krausz, Sigune Goldacker, Natalie Frede, Stephan Ehl, Sarah Salou, Ansgar Schulz, Shahrzad Bakhtiar, Maria Fasshauer, Oliver Hausmann, Anja-Dorothee Hüfner, Martin Stanulla, Anna Luijendijk, Faranaz Atschekzei, Georgios Sogkas, Ulrich Salzer, Baerbel Keller, Michele Proietti, Klaus Warnatz, Bodo Grimbacher

**Affiliations:** 1 Clinic of Rheumatology and Clinical Immunology, Center for Chronic Immunodeficiency (CCI), Medical Center, Faculty of Medicine, Albert-Ludwigs- University of Freiburg, Freiburg, Germany; 2 Institute for Immunodeficiency (IFI), Center for Chronic Immunodeficiency (CCI), Medical Center, Faculty of Medicine, Albert-Ludwigs-University of Freiburg, Freiburg, Germany; 3 FREEZE Biobank, Center for Biobanking, Albert-Ludwigs-University of Freiburg, Freiburg, Germany; 4 Department of Diagnostic and Interventional Radiology, Medical Center, Faculty of Medicine, Albert-Ludwigs-University of Freiburg, Freiburg, Germany; 5 Insitute of Medical Immunology, Berlin Institute of Health (BIH) & Charité University Medicine, Berlin, Germany; 6 Doctor’s Office for General Practice, Freiburg, Germany; 7 Division of Immunology, Faculty of Medicine and Health Sciences, University Medicine Oldenburg, Oldenburg, Germany; 8 Institute of Medical Genetics, Faculty of Medicine and Health Sciences, University Medicine Oldenburg, Oldenburg, Germany; 9 Department of Pediatrics, University Medical Center Ulm, Ulm, Germany; 10 Department for Pediatrics, Stem Cell Transplantation and Immunology, Goethe University Frankfurt/Main, Frankfurt, Germany; 11 Department for Pediatric Rheumatology and Immunology, St. Georg Clinic, ImmunDefektCentrum Leipzig (IDCL), Leipzig, Germany; 12 Loewenpraxis and Klinik St. Anna Lucerne, Lucerne, Switzerland; 13 Department for Infectious Diseases, University Clinic Hamburg-Eppendorf, Hamburg, Germany; 14 Department for Pediatric Hematology and Oncology, Hannover Medical School, Hannover, Germany; 15 Department for Pediatric Hematology and Oncology, Haukeland University Hospital Bergen, Bergen, Norway; 16 Department of Rheumatology and Immunology, Hannover Medical School, Hannover, Germany; 17 Cluster of Excellence RESIST (EXC 2155), Hannover Medical School, Hannover, Germany; 18 German Center for Infection Research, Satellite Center Freiburg (DZIF), Freiburg, Germany; 19 Centre for Integrative Biological Signalling Studies (CIBSS), Albert-Ludwigs University, Freiburg, Germany; 20 Cluster of Excellence 2155 to Hannover Medical School, Satellite Center Freiburg, Freiburg, Germany

**Keywords:** *NFKB1*, NF-κB1, Disease activity, Treatment, Score, *NFKB1*-DAS

## Abstract

*NFKB1* encodes the p105/p50 NF-κB1 transcription factor. Heterozygous mutations in *NFKB1* may lead to *NFKB1* insufficiency affecting multiple organ systems. This study aimed to develop an *NFKB1*-Disease-Activity-Score (*NFKB1*-DAS), serving as a tool in future clinical trials. Based on published phenotypic data from 179 patients with 77 distinct *NFKB1* variants, we drafted an initial score consisting of 42 parameters. This draft was subsequently analyzed in a multicenter cohort of 41 patients with 22 distinct pathogenic *NFKB1* variants. Using a Leave-One-Out (LOO) analysis (cutoff differential value: 23.07), we identified the 21 most relevant parameters to construct the final *NFKB1*-DAS. Calculation of the *NFKB1*-DAS in 31 *CTLA4*-deficient patients as a comparison cohort showed higher scores in *NFKB1*-insufficient patients (*p* = 0.03 (95% CI [-0.16, -0.01]) and the *NFKB1*-DAS correlated with hospitalization (*p* = 0.01; OR 2.07 (95% CI [1.18; 3.62]). However, given the optimism bias occurring when testing the score in the cohort it was developed with and the limited case numbers, validation of the *NFKB1*-DAS in an unrelated cohort remains essential. Analysis of the *NFKB1*-DAS in patients with variants of unknown significance (VUS) in *NFKB1* (*n* = 14) showed individual patients reaching high scores. Future studies could evaluate a possible utility of the score in assessing VUS pathogenicity. Ultimately, the proposed *NFKB1*-DAS represents a first step toward monitoring disease activity and treatment response in future clinical trials in *NFKB1 *insufficiency.

**Supplementary Information:**

The online version contains supplementary material available at https://doi.org/10.1007/s10875-026-02081-3.

## Introduction

*NFKB1* (Nuclear factor kappa B subunit 1) encodes the cytoplasmic precursor p105, processed to generate the NF-κB transcription factor subunit p50. The NF-κB family comprises the five members NF-κB1 (p105/p50), NF-κB2 (p100/p52), RelA (p65), RelB and c-Rel, being nearly ubiquitously expressed and forming a variety of homo- and heterodimers with activating or repressing functions depending on their combination. The NF-κB signaling system comprises the canonical (NF-κB1-dependent) and the non-canonical (NF-κB2-dependent) pathway. Canonical signaling is typically activated by inflammatory signals (such as TNF-α) *via* cell surface receptors, which triggers the release of mainly p50:RelA dimers from their cytoplasmic inhibitors (IκB), translocating to the nucleus to regulate their target genes including a variety of immune genes [1–3]. Reflecting extreme intolerance to inactivation, *NFKB1* exhibits a pLI of 1.0 and a LOEUF of 0.15, confirming severe constraint against loss-of-function variation [4]. 

Previous studies demonstrated that most pathogenic heterozygous *NFKB1* variants cause (haplo-) insufficiency (HI) of p105 and p50 which may result in a multiorgan disease, referred to here as *NFKB1* insufficiency, affecting respiratory, gastrointestinal, lymphatic system and the skin [5]. *NFKB1* insufficiency is the most common monogenic cause for primary antibody deficiency [5–7]. However, with 70%, the penetrance of proven pathogenic variants in *NFKB1* is incomplete and disease severity varies considerably even among carriers of the same mutation in *NFKB1.* [5, 8] The disease can progress chronically over many years and remains challenging to treat, resulting in diverse therapeutic strategies. In the cohort reported in this study, the predominant therapeutic approaches were immunoglobulin replacement, immunomodulatory therapy, or antibiotic prophylaxis of infections. Lacking large-scale, systematic treatment studies, the clinical presentation and the experience of the attending physician currently determine the preferred treatment. There is neither a targeted therapy to ‘cure’ *NFKB1* insufficiency, nor individualized therapies to manage unique clinical presentations or individual genetic variants [5]. 

Treatment studies on *NFKB1* insufficiency require an objective multi-organ composit score tool to assess the benefits of various therapeutic options. Applying global disease severity scores for inborn errors of immunity, such as common variable immunodeficiency (CVID) severity scores or scores addressing single organ manifestations like the Bhalla score for lung diseases or the NANO scale (Neurologic Assessment in Neuro-Oncology) for neurologic symptoms are not sufficient, since they are not specific enough for *NFKB1* insufficiency [9–14]. Our study processes previously known disease manifestations and newly collected data into a novel clinical tool, the *NFKB1*-DAS.

## Methods

### Analysis of previous *NFKB1* publications leads to the generation of a preliminary score based on published reports, expert knowledge and review of patients’ records

In July 2020 seven main publications were selected describing the *NFKB1*-associated disease phenotype in 179 patients with 77 distinct *NFKB1* variants classified as pathogenic or possibly pathogenic based on prediction tools or functional tests (Table S1). Based on the identified characteristic organ manifestations of *NFKB1* insufficiency a first score draft comprising 42 parameters was established (Table S2, Fig. S1 Part 1). Repeatedly reported symptoms and diagnoses were summarized in score parameters, partly using established scores like the CHAI morbidity score (from the ABACHAI study = Abatacept for CTLA4 HAploInsufficiency), the Hartmann score, an immunoglobulin replacement score, the Medical Research Council (MRC) Dyspnoea Scale, the Neurologic Assessment in Neuro-Oncology (NANO) Scale and the Disease Activity Score including C-reactive Protein (DAS-28-CRP) [13, 15–19] or experience from previous studies [22].

The *NFKB1*-DAS is indicated as a percentage value corresponding to the achieved score points relative to the maximum score value (File S4). The maximum possible score value results from the maximum number of points of all parameters with available data. Zero to three points can be achieved for each parameter (0 = not present, 1 = mild, 2 = moderate, 3 = severe).

### Score calculation according to the first score draft in a cohort of 41 patients with pathogenic variants in *NFKB1*

#### NFKB1 cohort and collection of clinical data

From October 2020 to February 2023, prospective and retrospective clinical data collection (going back to 2007) in a cohort of 41 patients with pathogenic variants in *NFKB1* was conducted, aiming to calculate the score according to the first score draft (Fig. S1 Part 2). Patients were recruited from clinical databases and eight centers in Germany and Switzerland and have a central European background in the vast majority [20, 21]. Our study cohort comprised 41 patients carrying 22 unique pathogenic *NFKB1* variants confirmed by functional in vitro assays to cause a protein defect or predicted to be pathogenic with a high probability (e.g. all truncations) (Tables 1 and 2, Table S3) [5, 6, 8, 23, 24]. In addition, 14 patients with 13 distinct variants of unknown significance (VUS) were collected. 

**Table 1:** In the study cohort, 35 unique variants in NFKB1 were identified

NFKB1- Variant ID	cDNA change	Protein change (* = stop)	AF	Variant ‘type’	ACMG classification	CADD PHRED	Funcionally tested in (PMID)	Result of functional test
Early truncation: haploinsufficiency (pathogenic)								
#1	c.159 + 1G > A	p.Ala40Glufs*10 (assumed)	NA	splice donor / out-of-frame delEx4	Pathogenic/likely pathogenic	33	26,279,205	pathogenic
#2	c.407 + 1G > A	p.Ile87Leufs*16 (assumed)	NA	splice donor / out-of-frame delEx6	Pathogenic/likely pathogenic	32	*	
#3	c.295 C > T	p.Gln99*	NA	nonsense	Pathogenic	38	34,473,196	pathogenic
#4	c.469 C > T	p.Arg157*	NA	nonsense	Pathogenic	38	34,473,196	pathogenic
#5	c.520_521insCTGA	p.Leu176*	NA	4 bp insertion / nonsense	Pathogenic	NA	34,473,196	pathogenic
#6	c.584_585delAG	p.Glu195Alafs*29	NA	2 bp deletion / frameshift	NA	NA	*	
#7	c.589delA	p.Ile197Serfs*55	NA	1 bp deletion / frameshift	Likely pathogenic	NA	*	
#8	c.634_656dupGTGGTGCGGCTCATGTTTACAGC	p.Phe220Trpfs*40	NA	23 bp duplication / frameshift	NA	NA	*	
#9	c.850 C > T	p.Arg284*	NA	nonsense	Pathogenic/likely pathogenic	43	34,473,196	pathogenic
#10	c.872delA	p.Asn291Metfs*141	NA	1 bp deletion / frameshift	Pathogenic	NA	34,473,196	pathogenic
#11	c.904dupT	p.Ser302Phefs*7	1.86e-6	1 bp duplication / frameshift	Pathogenic/likely pathogenic	NA	34,473,196	pathogenic
#12	c.1066 + 1G > C	p.Phe310Ilefs*76	NA	splice donor / out-of-frame delEx11	Pathogenic	33	*	pathogenic
#13	c.1012delT	p.Ser338Leufs*94	NA	1 bp deletion / frameshift	Pathogenic	NA	34,473,196	pathogenic
#14	c.1071_1074delAGAA	p.Glu358Lysfs*73	NA	4 bp deletion / frameshift	Pathogenic	NA	34,473,196	pathogenic
Central truncation: heterozygous loss-of precursor (pathogenic)								
#15	c.1361dupA	p.Asn454Lysfs*11	NA	1 bp duplication / frameshift	NA	NA	*	assumed to be pathogenic
#16	c.1365delT	p.Val456*	NA	1 bp deletion / nonsense	Pathogenic	NA	32,278,790	assumed to be pathogenic by fluorescence microscopy
#17	c.1819delG	p.Ala607Profs*3	NA	1 bp deletion / frameshift	NA	NA	*	assumed to be pathogenic
Late truncation (pathogenic)								
#18	c.2670delG	p.Ala891Glnfs*6	NA	1 bp deletion / frameshift	Likely pathogenic	NA	*	
#19	c.2455 C > T	p.Gln819*	NA	nonsense	Likely pathogenic****	37	*	
Early internal deletion: (p50) haploinsufficiency (pathogenic)								
#20	c.835 + 2T > G	p.Lys244_Asp279delinsAsn	NA	splice donor / in-frame delEx9	Pathogenic****	33	26,279,205	pathogenic
N-terminal half missense: DNA-binding defect (pathogenic)								
#21	c.169 C > T	p.Arg57Cys	NA	missense	VUS****	33	34,473,196	pathogenic
N-terminal half missense: loss-of-expression (pathogenic)								
#22	c.856T > A	p.Tyr286Asn	NA	missense	VUS****	288	34,473,196	pathogenic
N-terminal half missense: unclear defect (VUS)								
#23	c.150 A > C	p.Gln50His	NA	missense	VUS	243	41,202,999	**
#24	c.156 A > C	p.Lys52Asn	NA	missense	VUS	248		
#25	c.269 A > C	p.Tyr90Ser	NA	missense	VUS	279	36,105,815, 34,473,196	neutral
#26	c.307 A > G	p.Asn103Asp	6.20e-7	missense	VUS****	230	36,105,815	Unknown effect
#27	c.641G > A	p.Arg214Gln	NA	missense	VUS	34	34,473,196	Neutral (unclear in 36105815)
#28	c.646 A > G	p.Met216Val	NA	missense	VUS	243	34,473,196	Neutral (unclear in 36105815)
#29	c.734 C > T	p.Ala245Val	6.20e-7	missense	VUS****	282	34,473,196	Neutral (unclear in 36105815)
#30	c.736 C > A	p.Pro246Thr	NA	missense	VUS	255	34,473,196	neutral
C-terminal half missense: unclear defect (VUS)								
#31	c.1424 C > G	p.Ala475Gly	2.74e-4	missense	VUS****	4863	34,473,196	Unknown effect***
#32	c.1601G > A	p.Arg534His	4.73e-4	missense	Benign/likely benign	292	34,473,196	Unknown effect***
#33	c.1845G > T	p.Leu615Phe	2.45e-3	missense	Benign/likely benign	1572	34,473,196	Unknown effect***
#34	c.2548 C > T	p.Arg850Trp	1.00e-4	missense	VUS	32	34,473,196	Unknown effect***
#35	c.2634 A > T	p.Arg878Ser	1.36e-5	missense	VUS	1661	34,473,196	Unknown effect***

*AF* Allele frequency (from gnomAD 01-02-2026); ACMG classification from “ClinVar” (accessed on 2026-07-13) or “https://wintervar.wglab.org” or “https://www.ensembl.org/Multi/Tools/VEP/Ticket?tl=wRgldXpxRXdMh6qh” or PMID 32,278,790, *CADD PHRED*  Combined Annotation Dependent Depletion on PHRED (Phil’s Read Editor) scale, *NA*  Not reported in gnomAD

^*^not functionally tested in vitro but strongly predicted to be pathogenic; ^**^these two variants are neutral/inconspicuous when tested individually but have a pathogenic effect when cis-linked (Fliegauf et al. 2026, PMID 41202999), this was analyzed after data collection of the presented study. The NFKB1-DAS in the carrier was relatively high with 29%; ^***^reported in PMID 41,202,999 as neutral but performed test not sufficient for evaluating missense variants beyond amino acid 433 (common tests detect p50-specific functions only; p50 comprises the N-terminal half of p105 after cleavage). gnomAD reports high frequencies for variants #31-#34, usually in these cases no pathogenic pathomechanisms are found in functional tests. Variant #32 and #33 are assumed to present polymorphisms (likely benign) due to their high gnomAD frequency; ^****^low level of confidence of ACMG classification (single submitter or conflicting classifications of pathogenicity); VUS=Variant of unknown/uncertain significance

**Table 2:** Our study cohort includes 41 patients with pathogenic variants in NFKB1 and 14 patients with a VUS in NFKB1

	Patients with a pathogenic variant in NFKB1	Patients with a VUS in NFKB1
Total number of patients	41	14
Male (number/%)	16/39	9/64
Female (number/%)	25/61	5/36
Mean age at first survey date (years)	38	42
Median age at first survey date (years)	41	39
Minimal age at first survey date (years)	2	9
Maximum age at first survey date (years)	75	70
Deceased during the study period (number)	2	0
Age at death (years)	51 and 54	-

Cohort characteristics of 41 patients with pathogenic variants in NFKB1, analyzed for the development of the *NFKB1*-DAS, and 14 patients with VUS in NFKB1. Inclusion criteria were a heterozygous variant in *NFKB1* with a frequency in the healthy population of less than 1:100 on gnomAD (if reported) [27]. If a variant was predicted to be “neutral” based on the currently available functional in vitro tests, it was still considered as potentially pathogenic with a frequency of less than 1:100.000 (if reported) and symptoms typically associated with NF-κB1 dysregulation [5, 28]. Without signed consent, deceased, unavailable, with data for < 50% of score parameters or unaffected variant carriers were not included (last criteria chosen to minimize bias in the analysis due to the incomplete penetrance of *NFKB1* insufficiency). Carriers were considered unaffected when a maximum of one upper respiratory tract infection (URTI) occurred within the last six months and no other score parameter yielded any points. Cohort characteristics on the *CTLA4* comparison group can be found in the supplementary materials

Functional data for the evaluated *NFKB1* variants were synthesized from comprehensive benchmarking studies [6, 23, 25]. 

The information required for the score was collected based on patient interviews, using questionnaires (File S1 and File S2), and documented clinical data. In general, symptoms that are known to be immune-related were considered as associated with the underlying *NFKB1* insufficiency. To calculate the score parameter for programmed cell death protein one positive (PD-1^+^) T cells, 42 blood samples from 22 patients with pathogenic variants in *NFKB1* were analyzed by CyTOF (Fig. S2). For the later following comparison of the *NFKB1*-DAS in pathogenic vs. VUS patients, PD-1^+^ T cells were also calculated in 12 patients with VUS in *NFKB1* and analysed using Spearman correlation and negative binomial regression (Fig. 3).

#### Score calculation based on the initial score draft

Score values were calculated using Excel. One up to seven time points were achieved for each patient. The data used to determine the individual score parameters was not allowed to deviate by more than +/- 3 months from the survey date (resp. -6 months if it was the first survey date). The median time between the first and second time point was 9 months and 6 months between second and third time point.

### Reducing the number of score parameters using a LOO analysis

First, the four CT parameters were integrated into one reducing the total number to 39 parameters. For the following LOO analysis, score data of the first time point from 41 patients with pathogenic *NFKB1* variants was used. Only data from the first time point was used to minimize data gaps (not all patients had several assessments) and to avoid potential bias from longitudinal changes.

**Fig. 1:**
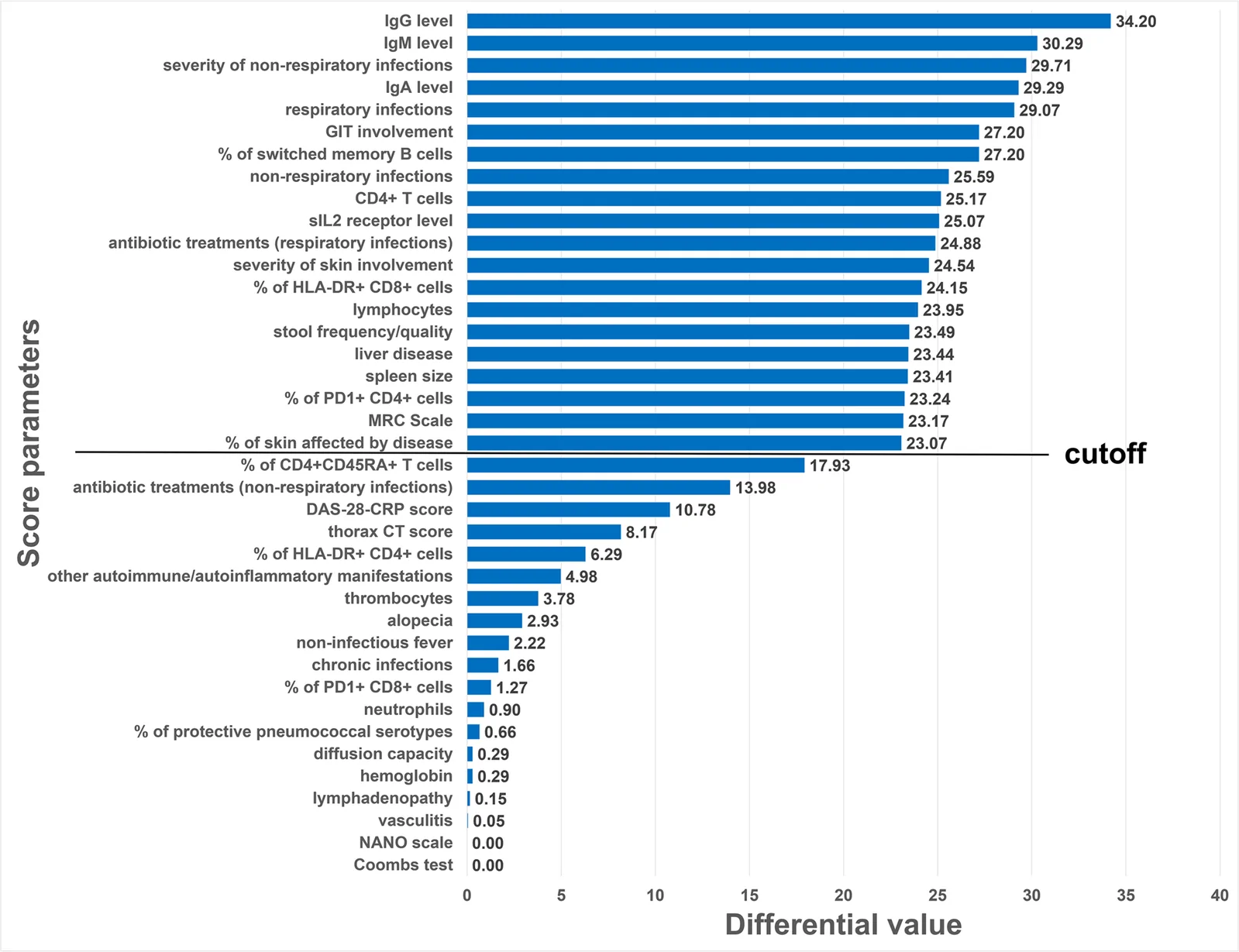
A leave-one-out analysis (LOO) defines a cut-off to identify the 21 most relevant score parameters. A LOO was used to assign a differential value to each parameter (described in Fig. 2a). Parameters were sorted according to this differential value. A cutoff was set between the two parameters with the largest difference in the differential values (see also Fig. 2b)

**Fig. 2:**
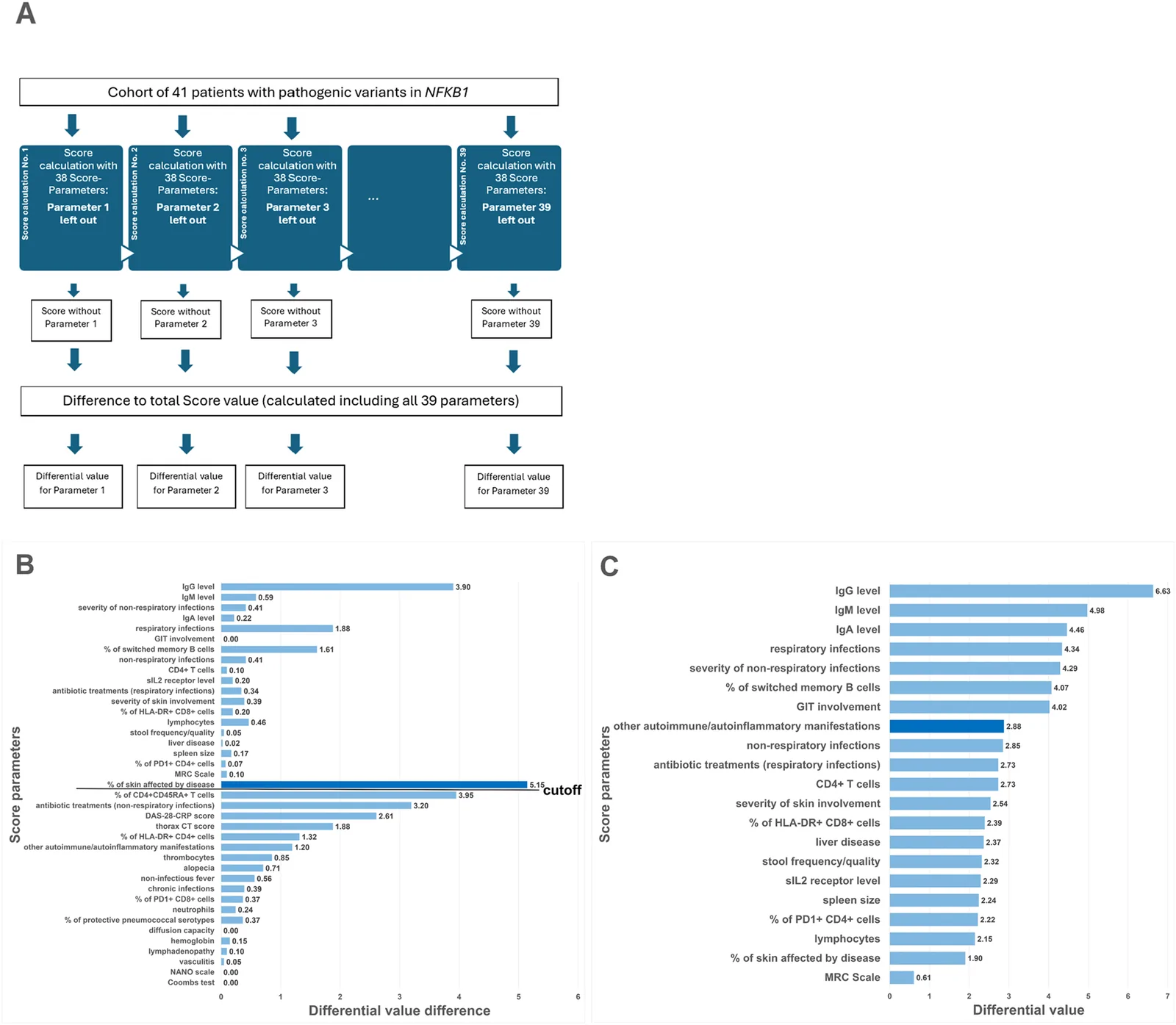
To perform the leave-one-out analysis (LOO), the score was calculated multiple times, each time omitting one of the 42 parameters. **a** A differential value identified in a LOO analysis defines the effect of each score parameter on the overall score. **b** A cutoff was set after the biggest differential value difference. **c** The parameter “autoimmune and autoinflammatory manifestations” shows a differential value in the middle range of all included parameters and was therefore also included in the score

To reduce the number of score parameters, we performed a LOO analysis at the parameter level (Fig. S1 Part 3). For each of the 39 parameters, the overall score was recalculated after omitting that parameter, while keeping the remaining 38 parameters unchanged. The resulting change in score was quantified as a differential value. Specifically, the differential value was defined as the mean squared difference between the original score and the score recalculated without that parameter across all patients. Parameters causing only small changes were considered less relevant and sequentially removed. The remaining parameters with the largest differential values were retained for the final score, reflecting their relative contribution to the overall score. The analysis was performed using R (Fig. 1 and Fig. 2a). The basic principles of this approach are described by e.g. Hastie et al. 2009 (p. 241ff.) or Wang et al., who called the method a Leave-One-Covariate-Out approach, but also included machine learning methods that were not part of the methods in this study [26, 27]. To identify a cutoff to decide which parameters to exclude from the score, parameters were sorted in descending order of their differential value. A cutoff was set between the two parameters with the largest difference in the differential values, as this reflected the largest drop of impact of parameters (Fig. 2). The parameter “other autoimmune/autoinflammatory manifestations” was considered separately since it summarizes multiple parameters, therefore it gains additional data points when other parameters are removed from the score. For instance, when “autoimmune vasculitis” was removed from the score because of a differential value below the cutoff, a vasculitis disease manifestation was scored in the parameter “other autoimmune/autoinflammatory manifestations”. When we repeated the analysis with the 20 parameters above the cut-off only plus “other autoimmune/autoinflammatory manifestations”, we obtained a differential value for the latter parameter in the mid-range, compared to other included parameters (Fig. 2). Therefore “other autoimmune/autoinflammatory manifestations” was included in the final *NFKB1*-DAS. The remaining 21 parameters form the final *NFKB1*-DAS presented here (Questionnaire in File S3, Score in File S4).

### Additional analysis with the final *NFKB1*-DAS investigating its performance

#### Analysis of the *NFKB1*-DAS in a control group of *CTLA4*-deficient patients

The *NFKB1*-DAS of 31 affected carriers with pathogenic variants in *CTLA4* (cytotoxic T-lymphocyte associated protein 4) (Tables S4, S5 and S7, all variants previously published [28–32]) was compared to the *NFKB1*-DAS in patients with pathogenic variants in *NFKB1* using a two-sided t test to have a first insight whether the score can reflect even subtle, potentially *NFKB1*-specific differences (Fig. S1 Part 4) [33]. The *CTLA4* cohort matched our *NFKB1* cohort very well regarding age and sex.

#### Analysis of hospitalization and correlation with the *NFKB1-DAS*

We chose hospitalization as a general marker to test the ability of the *NFKB1*-DAS to measure disease activity as a first proof-of-concept approach. *NFKB1*-DAS values of 41 patients with pathogenic variants in *NFKB1* were correlated with the number of days spent in hospital within the last 6 months using a hurdle model with negative binomial regression to account for overdispersion in our limited dataset (Fig. S1 Part 4). In all cases, the indication for hospitalization was classified as probably related to the underlying *NFKB1* insufficiency. Information on hospitalization was taken from medical records and patient questionnaires.

#### Calculation of the *NFKB1-*DAS in patients with VUS in *NFKB1*

During patient recruiting we found 14 patients with VUS in *NFKB1* (Table S3) and decided to calculate the *NFKB1*-DAS in these patients to examine differences in score values over time to see whether patients with pathogenic variants would show a more severe course of disease according to the *NFKB1*-DAS (Fig. S1 Part 4). Patients with VUS in *NFKB1* were examined and scored using the *NFKB1*-DAS as described before with a median time between first and second appointment of 6 months.

*The Online Repository* provides details on liver manifestations, comprehensive details on the publications analyzed for the development of the initial *NFKB1*-DAS draft, as well as the initial draft itself. A complete list of all patients included in the study—carriers of either *NFKB1* or *CTLA4* variants—is presented, alongside the specific *CTLA4* variants identified within the cohort. Table S6 summarizes the performance of the *NFKB1* patient cohort in the final *NFKB1*-DAS. Additionally, the questionnaires used for data collection in this study are included. File S3 (questionnaire) and File S4 (calculation procedure) can be used for future calculation of the *NFKB1*-DAS

## Results

### Data from a heterogenic *NFKB1* cohort with wide age distribution leads to the *NFKB1*-DAS comprising 21 score parameters

After performing the LOO analysis with data from our multicenter cohort, we present the *NFKB1*-DAS (File S4) consisting of the following parameters:

- Frequency of respiratory infections.
- frequency of antibiotic treatments (for respiratory infections).
- frequency of non-respiratory infections.
- severity of non-respiratory infections.
- MRC dyspnoea Scale.
- Stool frequency/quality.
- Gastrointestinal involvement.
- Severity of skin involvement.
- percentage (%) of skin affected by disease.
- Number of other autoimmune/autoinflammatory manifestations.
- Liver disease.
- Spleen size.
- Soluble Interleukin-2 (sIL2) receptor level.
- Immunoglobulin G (IgG) level.
- Immunoglobulin A (IgA) level.
- Immunoglobulin M (IgM) level.
- Lymphocytes.
- CD4 ^+^ T cells.
- percentage (%) of HLA-DR+ CD8 + cells.
- percentage (%) of PD-1^ +^ CD4^+^ cells.
- percentage (%) of switched memory B cells.

#### *NFKB1-*DAS in cohort of patients with pathogenic variants in *NFKB1*

Most patients with pathogenic variants in *NFKB1* showed hypogammaglobulinemia (81%) and splenomegaly (76%). Respiratory infections affected more than half of patients (60%). 40% of patients had skin manifestations (especially rosacea, psoriasis and vitiligo), but only 8% with systemic treatment. One third of patients (36%) suffered from gastrointestinal manifestations (36%) or liver involvement (32%) (Table S6, additional information on liver manifestations can also be found in the supplemental data).

#### Analysis of the PD-1 expression of CD4 + and CD8 + T cells in *NFKB1* insufficient patients

**Fig. 3:**
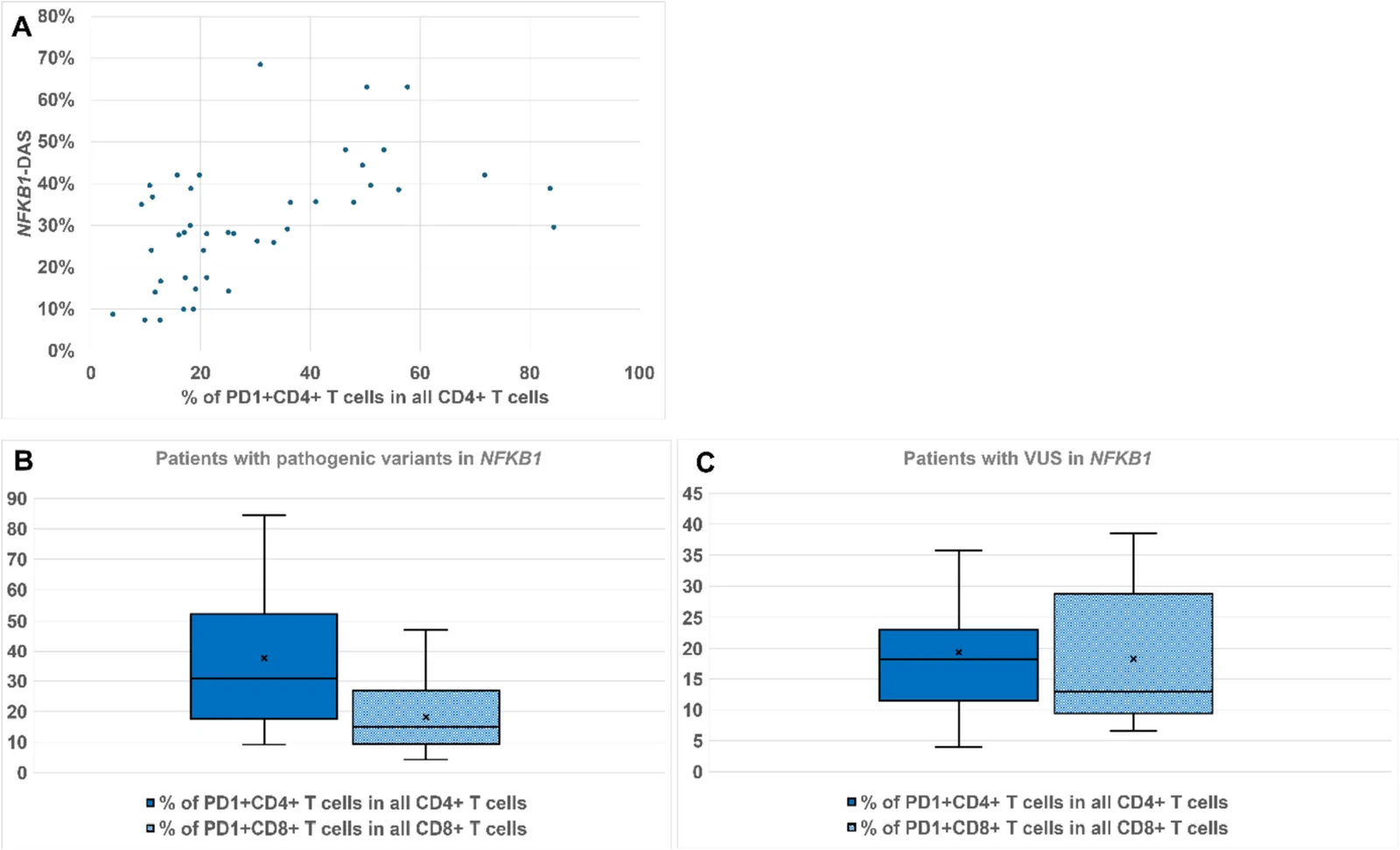
Patients with pathogenic variants in *NFKB1* show an increased level of PD-1^+ ^CD4^+^ T cells. **a** There is a positive correlation between the percentage of PD-1^+^ CD4^+^ T cells and the *NFKB1*-DAS. **b** In patients with pathogenic variants in *NFKB1*, the percentage of PD-1^+^ CD4^+^ T cells is higher than the percentage of PD-1^+^ CD8^+^ T cells, which was not seen in patients with VUS in *NFKB1* (**c**). The horizontal line shows median, x shows mean values

The PD-1 expression of CD4 + T cells was increased in 57% of patients. A Spearman correlation revealed an association between the *NFKB1*-DAS and CD4^+^ PD-1^+^ T cell levels in patients with pathogenic *NFKB1* variants (ρ = 0.73, *p* < 0.001; Fig. 3A). Consistent with this finding, a negative binomial regression demonstrated a 1.37-fold increase of the CD4 ^+^ PD-1^+^ T cell counts per 10-percent-point increase of the *NFKB1*-DAS (incidence rate ratio (IRR) 1.37; *p* < 0.001 (95% CI [1.18;1.60]). They show a higher percentage of PD-1^+^ CD4^+^ T cells (mean/median: 38%/31%) than PD-1^+^ CD8^+^ T cells (mean/median: 18%/15%) in patients with pathogenic variants in *NFKB1* (Fig. 3B), which we did not find in patients with VUS in *NFKB1* (mean/median of PD-1^+^ CD4^+^ T cells 19%/18%, mean/median of PD-1^+^ CD8^+^ T cells 18%/13%) (Fig. 3C).

#### Autoimmune manifestations are affecting a variety of organs and appear to disproportionately affect women

**Fig. 4:**
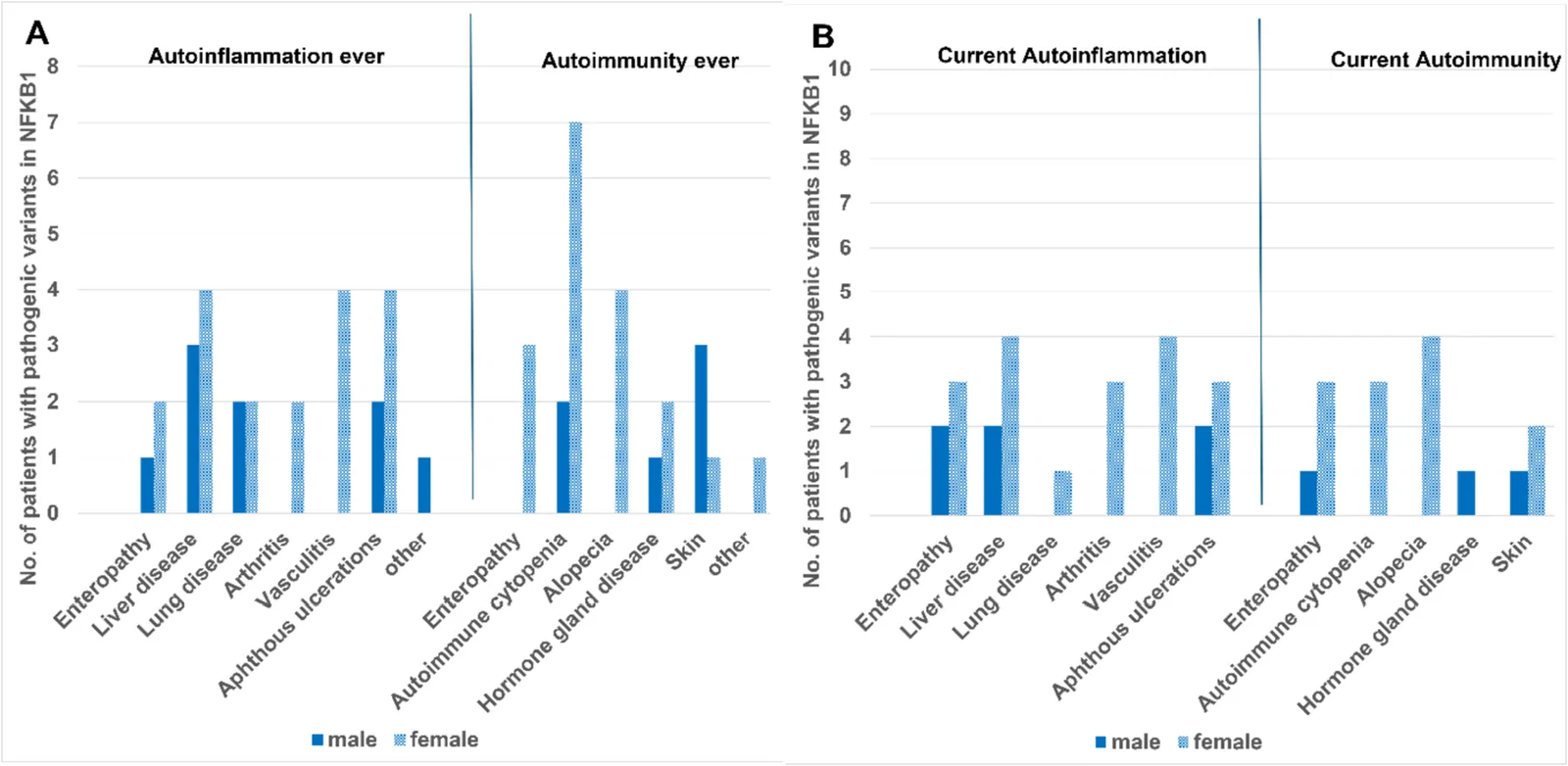
*NFKB1* patients show a variety of autoimmune and autoinflammatory manifestations, with a higher incidence in women. Female patients with pathogenic variants in *NFKB1* show a higher frequency of autoimmune and autoinflammatory manifestations, both in **a** lifetime and **b** current data (= at the study date). ”Other” in “Autoinflammation ever”: Bone marrow pathology associated with immunodeficiency. “Other” in “Autoimmunity ever”: Autoimmune encephalitis

54% of patients with pathogenic variants in *NFKB1* had at least one autoimmune or autoinflammatory disease not scored in other parameters. Female patients were affected more frequently. Manifestations included the gastrointestinal system, skin/hair, blood vessels, joints, liver, blood cells and lungs (Fig. 4).

### Additional analyses investigating the performance of the *NFKB1*-DAS

#### The *NFKB1-*DAS correlates with the hospitalization rate

**Fig. 5:**
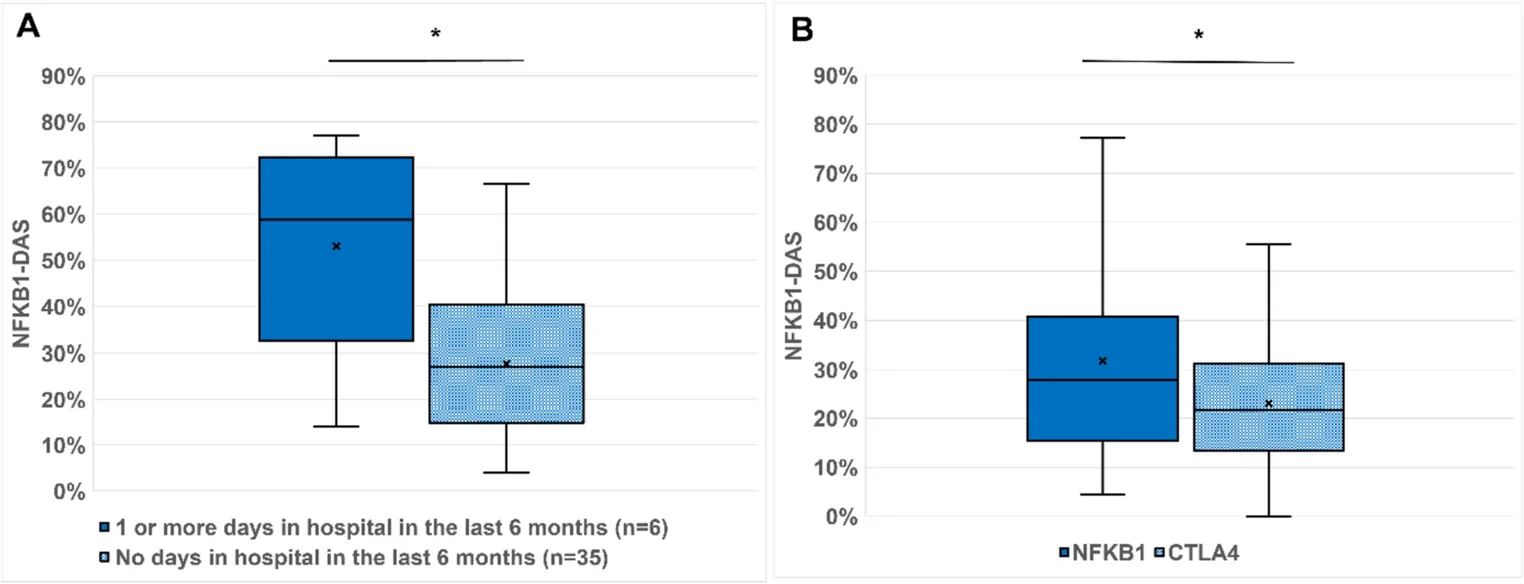
The *NFKB1*-DAS correlates with hospitalization and is higher in *NFKB1*insufficient patients than in patients with *CTLA4* deficiency. **a** The *NFKB1*-DAS correlates with hospitalization defined as at least one day spent in hospital in the last 6 months. **b** Patients with *NFKB1* insufficiency show higher *NFKB1*-DAS values than patients with *CTLA4* deficiency. The horizontal line shows median, x shows mean values

**Fig. 6:**
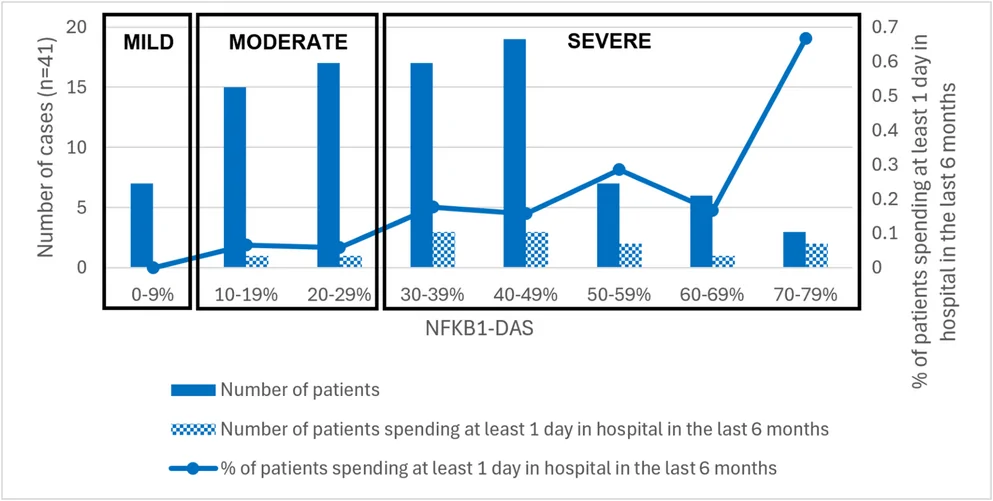
Based on the hospitalization rate, the *NFKB1*-DAS is classified in mild, moderate and severe categories. *NFKB1*-DAS values of our cohort of patients with pathogenic variants in *NFKB1* and the respective hospitalization rate led to a proposed classification of mild, moderate and severe *NFKB1*-DAS values

We performed a correlation of the *NFKB1*-DAS with the number of days spent in hospital in the last 6 months (Fig. S1 part 4). Patients were stratified into a hospitalized group (≥ 1 hospital day within the last 6 months) and a non-hospitalized group. A hurdle model showed that every 10-percent-point increase in the *NFKB1*-DAS score was associated with a doubling of the probability of hospitalization, defined as at least one day spent in hospital in the last 6 months (*p* = 0.01, OR 2.07, 95% CI [1.18; 3.62], mean/median *NFKB1*-DAS hospitalized group: 53%/58%, mean/median *NFKB1*-DAS non-hospitalized group: 28%/27%) (Fig. 5a). Based on a descriptive analysis of the hospitalization rate, we propose classifying *NFKB1*-DAS values into mild, moderate, and severe categories (Fig. 6): Mild defined as an *NFKB1*-DAS < 10% (those patients were not hospitalized in our cohort), moderate defined as an *NFKB1*-DAS < 30% (with a hospitalization rate of < 10% in our cohort), and severe defined as an *NFKB1*-DAS of 30% or higher (with a hospitalization rate of > 10% in our cohort).

#### Patients with pathogenic variants in *NFKB1* show higher *NFKB1-*DAS values than patients with variants in *CTLA4*

For a first approach testing whether the *NFKB1*-DAS differentiates between *NFKB1* insufficiency and other familiar phenotypes with incomplete penetrance, the scoring system was applied to a cohort of 31 patients with *CTLA4*-insufficiency as a control group (Fig. S1 Part 4, Table S4, S5, S7). *CTLA4*-deficient patients reached a mean *NFKB1*-DAS of 23% (median 22%) compared to 32% in the *NFKB1* cohort (median 28%) (Fig. 5b), which was significant with *p* = 0.03 (95% CI [-0.16, -0.01]).

#### Patients with pathogenic variants in *NFKB1* show an increasing *NFKB1*-DAS over time compared to patients with VUS in *NFKB1*

**Fig. 7:**
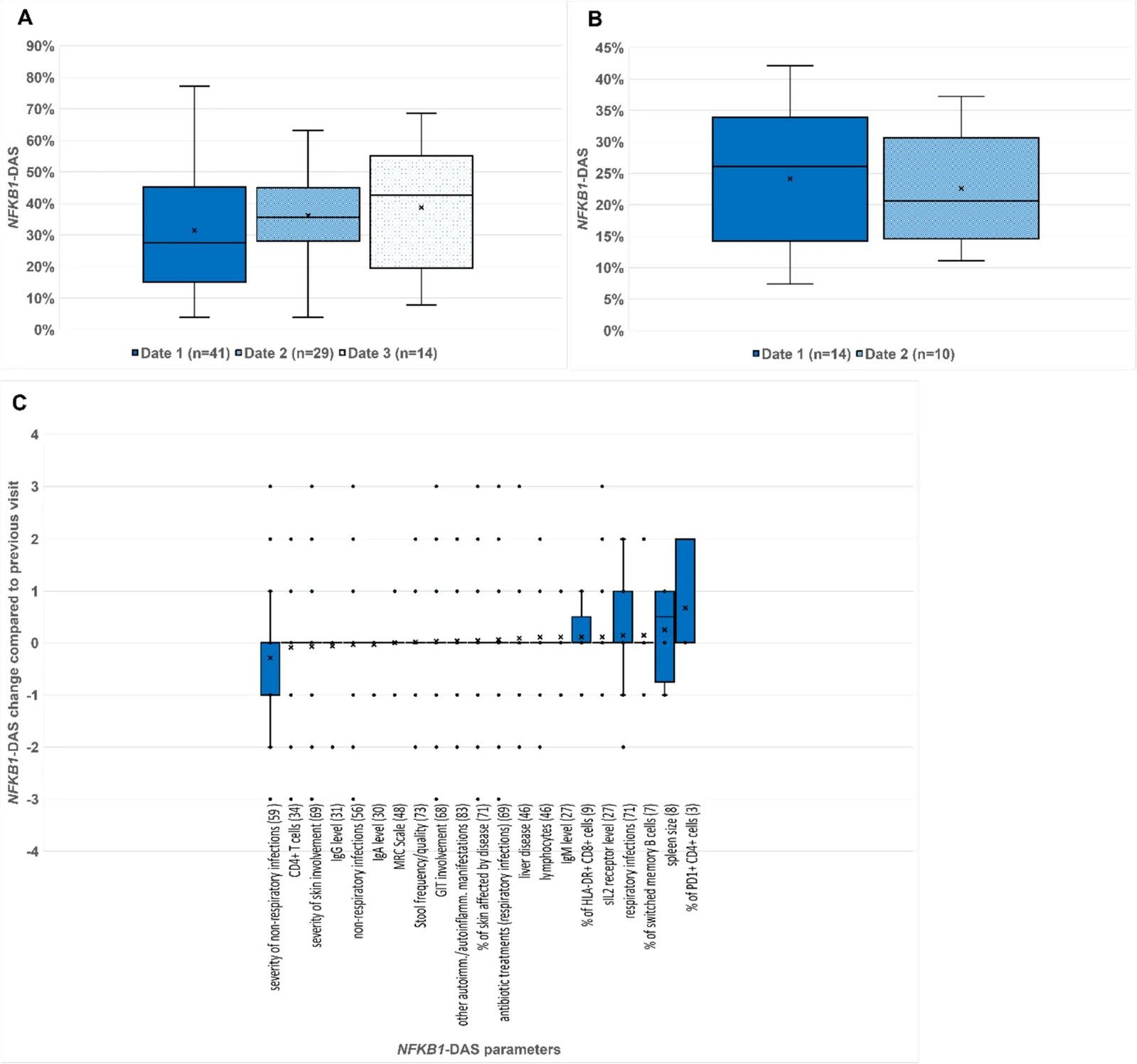
An analysis of the *NFKB1*-DAS over several study dates may indicate different disease progressions. **a **and** b** Patients with pathogenic variants in *NFKB1* show an increasing *NFKB1*-DAS over time (A), whereas patients with VUS in *NFKB1* show a decreasing *NFKB*1-DAS (B). (n = No. of patients). **c** Individual score parameters show divergent trends over time. In brackets: No. of calculations, one calculation = comparison of data from two study dates of a patient. The horizontal line shows median, x shows mean values

In patients with a pathogenic variant in *NFKB1*, mean and median *NFKB1*-DAS increased over time (Fig. 7a). In contrast, patients with a VUS in *NFKB1* had decreasing *NFKB1*-DAS scores over time (Fig. 7b). Longitudinal analysis of individual score parameters reveals divergent trends: Some parameters increase (e.g., spleen size or the percentage of PD-1⁺CD4⁺ T cells), while others decrease (e.g., severity of non-respiratory infections) or remain stable (Fig. 7c). These findings suggest that certain parameters may be more informative for tracking disease progression than others.

Almost all patients with a pathogenic variant in *NFKB1*, as well as patients with a VUS in *NFKB1* received systemic drug therapy (85% and 86% respectively). Children and adolescents < 18 years of age had a lower median *NFKB1*-DAS value (26%) than patients > 18 years of age (32%).

## Discussion

### Selecting score parameters

Following our literature analysis in 2020, a large meta-analysis has been published in 2024 by Fathi et al. [34] The results of this meta-analysis corresponded well with the parameters we selected for our *NFKB1*-DAS. The only manifestations not covered in the *NFKB1*-DAS are cardiovascular, neurological, and malignant manifestations, which were reported by Fathi et al. with a frequency of 0.6%, 1.7% and 5.6%, respectively. On purpose, we did not include malignancies in the *NFKB1*-DAS to avoid bias due to chemotherapy. Likewise, the IDDA (Immune Deficiency and Dysregulation Activity) 2.1 score also excludes malignancies from scoring [14]. 

### Recruiting patients

Since we recruited patients from immunology departments, there could be a bias regarding disease manifestations that are treated in other departments such as pyoderma. As the clinical picture of *NFKB1* insufficiency is primarily known in the context of immune dysregulation and therefore among immunologists, interdisciplinary collaboration and raising awareness of *NFKB1* insufficiency in other disciplines is essential to identify affected variant carriers.

### Data collection

*NFKB1* insufficiency is a rare disease which limits the number of patients in our study cohort. Data was collected during routine appointments which were often cancelled or postponed during the Covid-19 pandemic, both factors limiting a complete, systematic and broad data collection. We met these challenges by using a multicenter approach and extending our observation period to 2.5 years of prospective, as well as additional retrospective data collection. However, all presented analyses should be carefully interpreted considering the limited data.

### Systematic cohort analysis complements previous findings on the *NFKB1* phenotype

In our study we were able to perform a systematic extensive multicenter data collection on 41 patients with pathogenic variants in *NFKB1* as well as 14 patients with a VUS in *NFKB1* including data that have - to our knowledge - never been assessed before in *NFKB1* patients, such as PD-1^+^ T cell analysis. All variants have been reported before, but not all patients have been published with their full clinical picture. With the exception of a higher frequency of liver manifestations, our phenotypic data assessment fits the findings published in the largest *NFKB1* cohort to date by Lorenzini et al. [5] The overall higher proportion of women in our cohort (61%, Table 2) does not fully explain the higher proportion of women with autoimmune manifestations (median 71%; Fig. 4). Higher estrogen levels or incomplete X-chromosome inactivation could be contributing factors [35–38]. 

### Development of final *NFKB1*-DAS

The LOO approach allowed us to use all available patient data effectively, provided a transparent view of each parameter’s impact, and ultimately resulted in a more concise and interpretable score, even when starting from many parameters and a relatively small dataset. However, to mitigate the risk of overfitting inherent to this study design, these findings require validation in independent, heterogenous and ideally larger cohorts. Rare but severe disease manifestations may be underrepresented, as the LOO method emphasizes more frequent manifestations. At the same time, this focus ensures that the score reflects the typical and most observed phenotype and is feasible in clinical practice (one-page questionnaire in File S3). The cohort analysis combined with the LOO approach brings a significant improvement of methodology e.g. compared to the CHAI Morbidity score, developed solely based on expert knowledge [17]. Since the *NFKB1*-DAS can be calculated for the patient’s current disease state or cumulatively for the entire medical history, it can be used for prospective or retrospective studies. By calculating a score value in percent, we address the problem of missing data in clinical application. All parameters can be collected during regular clinical patient care. Also the PD-1^+^ CD4^+^ T cell analysis can be readily implemented in routine clinical laboratories (at a cost of approximately 15€) using a simple four-color whole-blood flow cytometry panel (CD45, CD3, CD4, and PD-1) on standard diagnostic instruments, making its translation into clinical practice technically feasible and relatively inexpensive.

### The *NFKB1*-DAS may uncover pathogenic effects of *NFKB1* variants classified as VUS

For the VUS identified in this study, no mechanistic insights into the pathomechanism of the resulting protein can be provided. This is a common limitation for VUS, where not only the clinical significance but often also the primary consequences at the protein function level remain unresolved. At the same time, ’neutral’ assessments in the currently available functional tests (which do not cover all protein functions) do not necessarily confirm a variant to be benign. On the contrary, particularly rare variants with strong variant effect predictions (VEPs), which were indistinguishable from controls in these assays, cannot be considered as necessarily non-pathogenic. We hypothesize that a high *NFKB1*-DAS value in patients carrying a VUS in *NFKB1* indicates NF-κB1 dysregulation due to a potentially pathogenic effect of the VUS. For instance, patients NFKB1-16 (carrying NFKB1-variant-30, c.736 C > A, p.Pro246Thr), NFKB1-20 (carrying NFKB1-variant-24, c.734 C > T, p.Ala245Val), and NFKB1-23 (carrying NFKB1-variant-29, c.269 A > C, p.Tyr90Ser), who all carry neutrally tested variants, have high score values of 36.84%, 35.71% and 42.11% (classified as severe according to our proposed scheme demonstrated in Fig. 6) at the first time point, respectively [6, 23]. Therefore, it could be worthwhile re-analyzing these variants for a functional defect as a first approach to test our hypothesis, followed by studying a possible correlation between the *NFKB1*-DAS and the pathogenicity of *NFKB1* variants in unrelated and larger cohorts. Moreover, the contribution of concomitant secondary e.g. (epi)-genetic factors should be considered. One example is patient NFKB1-37, who carries a VUS in *NFKB1* (NFKB1-variant-25, c.307 A > G, p.Asn103Asp) and a variant in *TNFRSF13B* (tumor necrosis factor receptor superfamily number 13B), encoding TACI (Transmembrane activator and CAML interactor) (c.579 C > A, p.Cys193*) who has a high *NFKB1*-DAS of 24%.

### Performance of the *NFKB1*-DAS

The *NFKB1*-DAS was higher in *NFKB1* insufficient patients than in patients with *CTLA4* deficiency, although *CTLA4*-deficient patients reached a mean *NFKB1*-DAS of 23%, which is due to shared phenotypic characteristics, such as hypogammaglobulinemia, autoimmune, respiratory and gastrointestinal manifestations [5, 33]. Our multi system composite *NFKB1*-DAS aims to offer a more specific representation of *NFKB1* insufficiency than previous scores developed for different or multiple diseases, such as the CHAI morbidity score or the IDDA 2.1 score, and to be a more suitable tool for treatment studies that rely on depicting the effects on specific disease manifestations as accurately as possible [14, 17]. The presented performance analyses (comparison with *CTLA4* cohort and hospitalization analysis) should be interpreted cautiously and perceived as proof-of-concept approaches, considering the limited sample size resulting in high variance and deviation from standard distribution. These analyses were also not adjusted for age, sex, or disease duration due to the limited data available. Conclusions regarding sensitivity and specificity of the *NFKB1*-DAS should not be drawn, instead, validation of the *NFKB1*-DAS should be performed in a follow-up study unrelated to the study cohort the score development was based on, to minimize bias. The proposed severity levels (Fig. 6), should be examined and further developed in these follow-up studies.

### Application of the *NFKB1*-DAS to guide medication strategies and track treatment success

The score data of this cohort demonstrates that disease activity increases with age even under medical treatment. This fits the long-assumed age-dependency of *NFKB1*-insufficiency and indicates that a disease-specific treatment is still missing. Follow-up studies should test this finding, since the number of patients with multiple score analyses was limited. *NFKB1* insufficiency can lead to hyperinflammatory disease similar to A20 haploinsufficiency, where a negative regulator of NF-κB is reduced and NF-κB signaling is overactive [39]. Whether anti-inflammatory drugs like TNF-α inhibitors are superior to other treatment options beyond individual cases is currently investigated in a worldwide *NFKB1* study using and validating the *NFKB1*-DAS. An important limitation in the assessment of complex chronic diseases, one that is also reflected in our score, is the unclear distinction between acute disease activity and chronic, permanent organ damage. Follow-up studies may be able to further refine the *NFKB1*-DAS in this regard.

## Conclusion

Increasing *NFKB1*-DAS values in affected *NFKB1* insufficient patients underline the urgent need for more efficient personalized treatment options. Our results pave the way for using the *NFKB1*-DAS as an objective tool for treatment studies in patients with *NFKB1*-insufficiency. Future studies should assess the ability of disease activity monitoring and potential diagnostic value of the proposed composite score.

## Supplementary Information

Below is the link to the electronic supplementary material.

Supplementary File (PDF 2.12 MB)

## Data Availability

The primary data are available from the corresponding author upon reasonable request.

## References

[1] Hayden MS, Ghosh S. NF-κB in immunobiology. Cell Res. 2011;21(2):223–44. 10.1038/cr.2011.13. PMC3193440 21243012

[2] Beinke S, Ley SC. Functions of NF-kappaB1 and NF-kappaB2 in immune cell biology. Biochem J. 2004;382(Pt 2):393–409. doi:10.1042/BJ20040544 PubMed PMID: 15214841; PubMed Central PMCID: PMC1133795. 10.1042/BJ20040544 PMC1133795 15214841

[3] Liu R, McEachin RC, States DJ. Computationally identifying novel NF-kappa B-regulated immune genes in the human genome. Genome Res. 2003;13(4):654–61. 10.1101/gr.911803 . PubMed PMID: 12654722; PubMed Central PMCID: PMC430162. PMC430162 12654722

[4] gnomAD Website zu NFKB1. 2023. Available from: https://gnomad.broadinstitute.org/gene/ENSG00000109320?dataset=gnomad_r2_1. [cited 2023 Aug 2].

[5] Lorenzini T, Fliegauf M, Klammer N, Frede N, Proietti M, Bulashevska A, et al. Characterization of the clinical and immunologic phenotype and management of 157 individuals with 56 distinct heterozygous NFKB1 mutations. J Allergy Clin Immunol. 2020 Apr;9. 10.1016/j.jaci.2019.11.051 . PubMed PMID: 32278790. PMC8246418 32278790

[6] Li J, Lei WT, Zhang P, Rapaport F, Seeleuthner Y, Lyu B, et al. Biochemically deleterious human NFKB1 variants underlie an autosomal dominant form of common variable immunodeficiency. J Exp Med. 2021;218(11):e20210566. 10.1084/jem.20210566 . PubMed PMID: 34473196; PubMed Central PMCID: PMC8421261. PMC8421261 34473196

[7] Tuijnenburg P, Lango Allen H, Burns SO, Greene D, Jansen MH, Staples E, et al. Loss-of-function nuclear factor κB subunit 1 (NFKB1) variants are the most common monogenic cause of common variable immunodeficiency in Europeans. J Allergy Clin Immunol. 2018;142(4):1285–96. .039 PubMed PMID: 29477724; PubMed Central PMCID: PMC6148345. 10.1016/j.jaci.2018.01.039 PMC6148345 29477724

[8] Fliegauf M, Bryant VL, Frede N, Slade C, Woon ST, Lehnert K, et al. Haploinsufficiency of the NF-κB1 Subunit p50 in Common Variable Immunodeficiency. Am J Hum Genet. 2015;97(3):389–403. PubMed PMID: 26279205; PubMed Central PMCID: PMC4564940. 10.1016/j.ajhg.2015.07.008 PMC4564940 26279205

[9] Mokhtari M, Shakeri A, Mirminachi B, Abolhassani H, Yazdani R, Grimbacher B, et al. Important Factors Influencing Severity of Common Variable Immunodeficiency. Arch Iran Med. 2016;19(8):544–50. doi:0161908/AIM.005 PubMed PMID: 27544362. 27544362

[10] Ameratunga R. Assessing Disease Severity in Common Variable Immunodeficiency Disorders (CVID) and CVID-Like Disorders. Front Immunol. 2018;9. 10.3389/fimmu.2018.02130. PubMed PMID: 30323807; PubMed Central PMCID: PMC6172311. PMC6172311 30323807

[11] Barbati F, Lodi L, Boscia S, Cortimiglia M, Calistri E, Quaranta F, et al. Monogenic Common Variable Immunodeficiency (Mo-CVID) Score for Optimizing the Genetic Diagnosis in Pediatric CVID Cohort. Eur J Immunol. 2025;55(3):e202451433. 10.1002/eji.202451433 . PubMed PMID: 40079712; PubMed Central PMCID: PMC11905875. PMC11905875 40079712

[12] Bhalla M, Turcios N, Aponte V, Jenkins M, Leitman BS, McCauley DI, et al. Cystic fibrosis: scoring system with thin-section CT. Radiology. 1991;179(3):783–8. 10.1148/radiology.179. .3.2027992 PubMed PMID: 2027992. 2027992

[13] Nayak L, DeAngelis LM, Brandes AA, Peereboom DM, Galanis E, Lin NU, et al. The Neurologic Assessment in Neuro-Oncology (NANO) scale: a tool to assess neurologic function for integration into the Response Assessment in Neuro-Oncology (RANO) criteria. Neuro Oncol. 2017;19(5):625–35. 10.1093/neuonc/nox. 029 PubMed PMID: 28453751; PubMed Central PMCID: PMC5464449. PMC5464449 28453751

[14] Seidel MG, Tesch VK, Yang L, Hauck F, Horn AL, Smolle MA, et al. The Immune Deficiency and Dysregulation Activity (IDDA2.1 ’Kaleidoscope’) Score and Other Clinical Measures in Inborn Errors of Immunity. J Clin Immunol. 2022;42(3):484–98. 10.1007/s10875-021-01177-2 . PubMed PMID: 34797428; PubMed Central PMCID: PMC9016022. PMC9016022 34797428

[15] Website des MRC. 2023. Available from: https://www.ukri.org/councils/mrc/facilities-and-resources/find-an-mrc-facility-or-resource/mrc-dyspnoea-scale/. [cited 2023 Aug 2].

[16] Fransen J, Welsing P, De Keijzer R, Van Riel P. Disease Activity Scores Using C-reactive, Protein: CRP may Replace ESR in the Assess-ment of RA Disease Activity. 2004. Available from: https://scholar.google.com/scholar_lookup?title=Disease%20activity%20scores%20using%20C-reactive%20protein%3A%20CRP%20may%20replace%20ESR%20in%20the%20assessment%20of%20RA%20disease%20activity&author=J%20Fransen&author=PMJ%20Welsing&author=RMH%20de%20Keijzer&author=PLCM%20van%20Riel&publication_year=2003&journal=Ann%20Rheum%20Dis&volume=62(Suppl.%201)&pages=151. [cited 2023 Aug 2]

[17] Krausz M, Uhlmann A, Rump IC, Ihorst G, Goldacker S, Sogkas G, et al. The ABACHAI clinical trial protocol: Safety and efficacy of abatacept (s.c.) in patients with CTLA-4 insufficiency or LRBA deficiency: A non controlled phase 2 clinical trial. Contemp Clin Trials Commun. 2022;30:101008. 10.1016/j.conctc.2022.101008 . PubMed PMID: 36262801; PubMed Central PMCID: PMC9573884. PMC9573884 36262801

[18] Meerburg JJ, Hartmann IJC, Goldacker S, Baumann U, Uhlmann A, Andrinopoulou ER, et al. Analysis of Granulomatous Lymphocytic Interstitial Lung Disease Using Two Scoring Systems for Computed Tomography Scans—A Retrospective Cohort Study. Front Immunol. 2020;11:589148. 10.3389/fimmu.2020.589148 . PubMed PMID: 33193417; PubMed Central PMCID: PMC7662109. PMC7662109 33193417

[19] Agarwal S, Cunningham-Rundles C. Treatment of hypogammaglobulinemia in adults: a scoring system to guide decisions on immunoglobulin replacement. J Allergy Clin Immunol. 2013;131(6):1699–701. 10.1016/j.jaci.2013.01.036 . PubMed PMID: 23518142; PubMed Central PMCID: PMC4102312. PMC4102312 23518142

[20] Caballero-Oteyza A, Crisponi L, Peng XP, Yauy K, Volpi S, Giardino S, et al. GenIA, the Genetic Immunology Advisor database for inborn errors of immunity. J Allergy Clin Immunol. 2023;S0091–6749(23):01513–0. 10.1016/j.jaci.2023.11.022 . PubMed PMID: 38040041. 38040041

[21] The ESID Registry. Available from: https://cci-esid-reg.uniklinik-freiburg.de/EERS/login/auth. [cited 2024 Aug 5].

[22] von Spee-Mayer C, Echternach C, Agarwal P, Gutenberger S, Soetedjo V, Goldacker S, et al. Abatacept Use Is Associated with Steroid Dose Reduction and Improvement in Fatigue and CD4-Dysregulation in CVID Patients with Interstitial Lung Disease. J Allergy Clin Immunology: Pract. 2021;9(2):760–e77010. 10.1016/j.jaip.2020.10.028. 33223097

[23] Fliegauf M, Kinnunen M, Posadas-Cantera S, Camacho-Ordonez N, Abolhassani H, Alsina L, et al. Detrimental NFKB1 missense variants affecting the Rel-homology domain of p105/p50. Front Immunol. 2022;13:965326. 10.3389/fimmu.2022.965326 . PubMed PMID: 36105815; PubMed Central PMCID: PMC9465457. PMC9465457 36105815

[24] Lougaris V, Patrizi O, Baronio M, Tabellini G, Tampella G, Damiati E, et al. NFKB1 regulates human NK cell maturation and effector functions. Clin Immunol. 2017;175:99–108. 10.1016/j.clim.2016.11.012 . PubMed PMID: 27923702. 27923702

[25] Fliegauf M, Nitschke K, Mrovecova P, Posadas-Cantera S, Camacho-Ordonez N, Warnatz K, et al. Compound heterozygous NFKB1 missense variants in cis associated with immunodeficiency. Clin Immunol. 2026;282:110637. 10.1016/j.clim.2025.110637 . PubMed PMID: 41202999. 41202999

[26] Hastie T, Tibshirani R, Friedman J. The Elements of Statistical Learning. New York, NY: Springer; 2009. (Springer Series in Statistics). Available from: http://link.springer.com/10.1007/978-0-387-84858-7. https://doi.org/10.1007/978-0-387-84858-7. [cited 2025 Aug 24].

[27] Wang X, Zhou Y, Hooker G. Targeted Learning for Variable Importance.

[28] Schubert D, Bode C, Kenefeck R, Hou TZ, Wing JB, Kennedy A, et al. Autosomal-dominant immune dysregulation syndrome in humans with CTLA4 mutations. Nat Med. 2014;20(12):1410–6. 10.1038/nm.3746 . PubMed PMID: 25329329; PubMed Central PMCID: PMC4668597. PMC4668597 25329329

[29] Rojas-Restrepo J, Caballero-Oteyza A, Huebscher K, Haberstroh H, Fliegauf M, Keller B, et al. Establishing the Molecular Diagnoses in a Cohort of 291 Patients With Predominantly Antibody Deficiency by Targeted Next-Generation Sequencing: Experience From a Monocentric Study. Front Immunol. 2021;12:786516. 10.3389/fimmu.2021.786516 . PubMed PMID: 34975878; PubMed Central PMCID: PMC8718408. PMC8718408 34975878

[30] Egg D, Rump IC, Mitsuiki N, Rojas-Restrepo J, Maccari ME, Schwab C et al. Therapeutic options for CTLA-4 insufficiency. J Allergy Clin Immunol. 2021;S0091-6749(21)00891-5. 10.1016/j.jaci.2021.04.039 PubMed PMID: 34111452. 34111452

[31] Rojas-Restrepo J, Sindram E, Zenke S, Haberstroh H, Mitsuiki N, Gabrysch A, et al. Functional Relevance of CTLA4 Variants: an Upgraded Approach to Assess CTLA4-Dependent Transendocytosis by Flow Cytometry. J Clin Immunol. 2023;43(8):2076–89. 10.1007/s10875-023-01582-9 . PubMed PMID: 37740092; PubMed Central PMCID: PMC10661720. PMC10661720 37740092

[32] Ochoa S, Abers MS, Rosen LB, Rump A, Howe K, Lieberman JA, et al. Management and outcome of COVID-19 in CTLA-4 insufficiency. Blood Adv. 2023;7(19):5743–51. 10.1182/bloodadvances.2023010105 . PubMed PMID: 37406177; PubMed Central PMCID: PMC10539877. PMC10539877 37406177

[33] Schwab C, Gabrysch A, Olbrich P, Patiño V, Warnatz K, Wolff D, et al. Phenotype, penetrance, and treatment of 133 cytotoxic T-lymphocyte antigen 4-insufficient subjects. J Allergy Clin Immunol. 2018;142(6):1932–46. 10.1016/j.jaci.2018.02.055 . PubMed PMID: 29729943; PubMed Central PMCID: PMC6215742. PMC6215742 29729943

[34] Fathi N, Nirouei M, Salimian Rizi Z, Fekrvand S, Abolhassani H, Salami F, et al. Clinical, Immunological, and Genetic Features in Patients with NFKB1 and NFKB2 Mutations: a Systematic Review. J Clin Immunol. 2024;44(7):160. 10.1007/s10875-024-01763-0. 38990428

[35] Zhang Y, Li X, Gibson A, Edberg J, Kimberly RP, Absher DM. Skewed allelic expression on X chromosome associated with aberrant expression of XIST on systemic lupus erythematosus lymphocytes. Hum Mol Genet. 2020;29(15):2523–34. 10.1093/hmg/ddaa131 . PubMed PMID: 32628254. 32628254

[36] Tukiainen T, Villani AC, Yen A, Rivas MA, Marshall JL, Satija R, et al. Landscape of X chromosome inactivation across human tissues. Nature. 2017;550(7675):244–8. 10.1038/nature24265 . PubMed PMID: 29022598; PubMed Central PMCID: PMC5685192. PMC5685192 29022598

[37] Youness A, Miquel CH, Guéry JC. Escape from X Chromosome Inactivation and the Female Predominance in Autoimmune Diseases. Int J Mol Sci. 2021;22(3):1114. 10.3390/ijms22031114 . PubMed PMID: 33498655; PubMed Central PMCID: PMC7865432. PMC7865432 33498655

[38] Zhang H, Zhang Y, Wang YF, Morris D, Hirankarn N, Sheng Y, et al. Meta-analysis of GWAS on both Chinese and European populations identifies GPR173 as a novel X chromosome susceptibility gene for SLE. Arthritis Res Ther. 2018;20(1):92. 10.1186/s13075-018-1590-3 . PubMed PMID: 29724251; PubMed Central PMCID: PMC5934841. PMC5934841 29724251

[39] Yu MP, Xu XS, Zhou Q, Deuitch N, Lu MP. Haploinsufficiency of A20 (HA20): updates on the genetics, phenotype, pathogenesis and treatment. World J Pediatr. 2020;16(6):575–84. 10.1007/s12519-019-00288-6. 31587140

