# Supplementary material for: A multi-composite disease activity score (NFKB1-DAS) for NFKB1 insufficiency
Source: J Clin Immunol. 2026 Oct 19;46(1):96. doi: 10.1007/s10875-026-02081-3 (PMC13680531; doi:10.1007/s10875-026-02081-3)
Supplement: Supplementary file 1 [file 10875_2026_2081_MOESM1_ESM.pdf]

Supplemental data

Liver manifestations in NFKB1 cohort

One patient of our cohort of pathogenic variants in *NFKB1* had viral hepatitis (and one patient carrying a VUS in *NFKB1*). Liver biopsy findings were available for five patients with pathogenic variants in *NKFB1.* Four of them showed strong (1 patient) or possible (3 patients) signs of NRH, one showed regenerative hyperplasia without a nodular aspect. In three biopsies, inflammation of the liver tissue was detected. The lymphocytic infiltration was T-cell-dominated in all cases. In two cases the subgroups of T cells were further analyzed, one case showed predominant $CD4^{+}$ T cells and one case predominant $CD8^{+}$ T cells.

NFKB1 variant types

Many reported *NFKB1* variants cluster within key functional domains of the p105/p50 protein, particularly the highly conserved N-terminal Rel-homology domain (RHD; aa 34–363), which is essential for DNA binding, dimerization, and IκB interaction. Other critical regions include the nuclear localization sequence (NLS; aa 363–375), the glycine-rich region (GRR; aa 375–400) required for p105 processing, the C-terminal ankyrin repeat domain (ARD; aa 543–751), and the death domain (DD; aa 818–893). "Early" N-terminal truncations likely yield non-functional, rapidly degraded proteins leading to p105/p50 haploinsufficiency, whereas central truncations bypass the precursor stage ("precursor-skipping") to potentially generate p50-like molecules (if expressed at all). While C-terminal truncations are rare, premature stop codons are generally assumed to cause protein deficiency via nonsense-mediated mRNA decay (NMD). Similarly, most of the known pathogenic missense variants in the p50 subunit cause protein loss, whereas stable but functionally impaired variants remain rare, and the impact of C-terminal p105-specific missense alterations remains unclear. (1,2)

Methodology of functional testing of *NFKB1* variants

Standard *in vitro* characterization workflows for both major studies predominantly relied on transient overexpression models. This approach operates under the assumption that variants drive the expression of mutant proteins, thereby bypassing potential nonsense-mediated mRNA decay (NMD) mechanisms. Typically, mutant cDNAs are cloned into expression vectors and transfected into host cell lines to evaluate downstream functional consequences, where any significant deviation from the wild-type control is interpreted as a protein defect indicating a pathogenic effect. Fliegauf et al. utilized a transient overexpression system in human embryonic kidney (HEK293T) cells using N-terminally EGFP-tagged wild-type or mutant p105

and p50 constructs. (1,3,4) Subcellular localization was evaluated via fluorescence microscopy, identifying deleterious variants by characteristic sub-nuclear, spot-like accumulations indicative of accelerated protein decay. Western blotting was employed to analyze overall protein stability and the efficiency of proteasomal processing of the p105 precursor. Downstream functional impacts were assessed via Electrophoretic Mobility Shift Assays (EMSA) to measure DNA-binding capacity, alongside a fluorescence-based reporter competition assay to monitor the attenuation of RelA-mediated transcriptional activation. To map the variants' interactome and resolve protein-protein interactions beyond transient systems, Affinity Purification-Mass Spectrometry (AP-MS) and BioID were performed on inducible stable cell lines.

In contrast, Li et al. established a biochemical framework by generating an *NF-κB1*-deficient HEK293T cell line via CRISPR-Cas9 technology to eliminate interference from endogenous proteins. (2) Following the transfection of mutant cDNAs into these knockout cells, protein expression and proteolytic cleavage were systematically evaluated by Western blotting. To selectively quantify p50-specific transcriptional activity and identify potential dominant-negative effects via 1:1 co-transfection assays, the authors developed an elegant luciferase reporter assay utilizing a homodimerization-defective p65 mutant (S276A) to prevent false-positive signals from p65 homodimers. Additionally, an exon-trapping assay was conducted in COS-7 cells to verify the generation of abnormal transcripts for potential splice-site variants. Functional testing using primary patient cells remains less common. Primary assays frequently yield inconclusive results due to confounding clinical variables, such as underlying cytopenia, and the practical limitations of requiring repeated blood sampling, which confounds direct patient-to-healthy-donor comparisons.

Table S1: Seven main publications on the NF-κB1 phenotype were analyzed to identify characteristic organ manifestations.

**Publication No.**		**1**	**2**	**3**	**4**	**5**	**6**	**7**	
**PMID**		**32278790**	**29477724**	**26279205**	**28115215**	**27555455**	**27365489**	**27338827**	
**Authors of publication**		**Lorenzini et al.**	**Tuijnenburg et al.**	**Fliegauf et al.**	**Kaustio et al.**	**Lougaris et al.**	**Schipp et al.**	**Boztug et al.**	
**No. of individual patients**	**In all publications: 179**	**157**	**16**	**20**	**14**	**2**	**2**	**1**	
**No. of distinct *NFKB1* variants**	**In all publications: 77**	**56**	**16**	**3**	**3**	**2**	**2**	**1**	
**Category**	**Symptom**								**included in score**
Autoimmune and autoinflammatory manifestations	total	Autoimmunity: 58% Autoinflammation: 30%	10 (63%)	4 (20%)	7+ (50%+)	1 (50%)	2 (100%)	1 (100%)	yes
Blood and immune system	total	x	x	4 (20%)	10 (71%)	1 (50%)	2 (100%)	1 (100%)	yes
	Antibody deficiency	89%	x	x	10 (71%)	1 (50%)	2 (100%)	1 (100%)	

	Poor vaccine response				7 (50%)	1 (50%)	2 (100%)	1 (100%)	
	Altered B lymphocyte subgroups, e.g. reduced class switched B cells, CD21lo, plasma cells		x		10 (71%)	1 (50%)	1 (50%)	1 (100%)	
	Altered T lymphocyte subgroups, e.g. reduced naive CD4+ T cells and impaired function		x		x		2 (100%)	1 (100%)	
	Lack of calcium and Vitamin D						1 (50%)		no
Respiratory infections	total	x	15 (94%)	11 (55%)	10 (71%)	2 (100%)	2 (100%)	1 (100%)	yes

	Infections of the upper airways	83%	10	7	7 (50%)	2 (100%)	2 (100%)	1 (100%)	
	Infections of the lower airways	59%	14	6		2 (100%)	2 (100%)		
Non-respiratory infections	total	Gastrointestinal: 29%, skin: 38% and other	x	3 (15%)	x		2 (100%)	1 (100%)	yes
Lung	total	GLILD: 7% Brochiectasis: 26%	11+ (69% +)	8 (40%)	x		2 (100%)	1 (100%)	yes
	GLILD	7%	2	x					
	Bronchiectasis	26%	11	2			1 (50%)		
	other (COPD, unspecific, Lung fibrosis, Asthma)		Fibrosis :4 Asthma: 1	7	x		2 (100%)	1 (100%)	
Skin	total	x		2 (10%)	x		1 (50%)		yes
Liver	total	24%	7 (44%)	4 (20%)				1 (100%)	yes

Spleen	Splenomegaly or Splenectomy	Splenomegaly: 49% Splenectomy: 12%	11 (69%)	1 (5%)			x	1 (100%)	yes
Lymphoproliferation and/or Lymphadenopathy	total	35%	5 (31%)	2 (10%)			x	1 (100%)	yes
Gastrointestinal Manifestations	total	Autoimmune: 14%, celiac-like disease: 9%, IBD-like disease: 6%, atrophic gastritis: 5% 8%	4 (25%)	2 (10%)	6 (43%)		2 (100%)		yes
Joints	Arthralgia, Arthritis, Enthesiopathy	Arthritis: 10%, osteopenia: 13%, enthesiopathy			4 (29%)			1 (100%)	yes
Malignant Neoplasia	hematologic	11%	4 (25%)						no
	Solid	5%	2 (13%) (parathyroid adenoma,	2 (10%) (1 squamou					no

			breast cancer)	s cell carcinoma, 1 Adenoca of the lung)					
Benign Neoplasia	total				1 (7%) (Kidney tumor)	1 (50%) (gastric adenoma)			no
Nephrologic diesases	unspecific				1 (7%) (one-sided rudimentary kidney)		1 (50%) (impaired kidney function)		no
Neurologic manifestations	total	14%					1 (50%)		yes
Cardiovascular diseases	total	arteriosclerotic vasculopathy (11%), congenital heart defect (ventricular septal defect,		2 (10%) (1 with ischemic heart disease, 1 with aortic stenosis)					no

		mitral valve defect and patent ductus arteriosus, 3%), atrial arrhythmia (3%) and myocarditis (2%)								

Table S2: Based on the literature, a first draft of the NFKB1-DAS contained 42 parameters.

Criteria	Score value	Description
**Blood**		
Current IgG level in mg/dl(5)	None (0)	>/= 700
	Mild (1)	400-699
	Moderate (2)	200-399
	Severe (3)	<200 or IgG replacement
Current IgA level in mg/dl	None (0)	>70(6)
	Mild (1)	10-70
	Severe (3)	<10
Current IgM level in mg/dl	None (0)	>40(6)
	Mild (1)	31-40
	Moderate (2)	21-30
	Severe (3)	</=20
Current % of protective pneumococcal serotypes	None (0)	>60%
	Mild (1)	41-60%
	Moderate (2)	21-40%
	Severe (3)	</=20%
Current Hb in g/dl (for adults)(7)	None (0)	>10
	Mild (1)	9.1-10
	Moderate (2)	7.1-9
	Severe (3)	</= 7 or pancytopenia
Current Coombs Test	0	DCT negative
	3	DCT positive
Current number of thrombocytes (platelets/µl)	None (0)	>/=100.000
	Mild (1)	<100.000
	Moderate (2)	<50.000
	Severe (3)	<10.000
Current number of neutrophils/µl	None (0)	>1500(8)
	Mild (1)	>1000
	Moderate (2)	>500
	Severe (3)	</=500
Current number of lymphocytes/µl	None (0)	>/=1000
	Mild (1)	<1000
	Moderate (2)	<750

	Severe (3)	<500
Current number of CD4+ T cells/µl(7)	None (0)	>500
	Mild (1)	351-500
	Moderate (2)	200-350
	Severe (3)	<200
Current Naive (CD4+CD45RA+) T cell %(7)	None (0)	>20%
	Mild (1)	15-20%
	Moderate (2)	10-14.9%
	Severe (3)	<10%
Current CD4-T cell activation (% of HLA-DR+ CD4+ cells in all CD4+ cells)(8)	None (0)	< 20%
	Mild (1)	<30%
	Moderate (2)	<40%
	Severe (3)	>/= 40%
Current CD8-T cell activation (% of HLA-DR+ CD8+ cells in all CD8+ cells) *(Coop. with Ulrich Salzer)*	None (0)	< 35%
	Mild (1)	<40%
	Moderate (2)	<60%
	Severe (3)	≥60%
Current PD1 expression on CD4+ cells (% of CD4+PD1+ in all CD4+) *(improved parameter from CHAI Morbidity Score*(7)*)*	None (0)	<20%
	Mild (1)	<30%
	Moderate (2)	<40%
	Severe (3)	>/= 40%
Current PD1 expression on CD8+ cells (% of CD8+PD1+ in all CD8+)	None (0)	<20%
	Mild (1)	<30%
	Moderate (2)	<40%
	Severe (3)	>/= 40%
Current percentage of switched memory B cells in all B cells (% of CD19/20+,IgM-,CD27+ in all B cells)(7)	None (0)	>/= 6%
	Mild (1)	<6%
	Moderate (2)	<4%
	Severe (3)	< 2%
**Lymphadenopathy**		
Current number of enlarged (>/=1cm(9)) non-contiguous lymph node groups (evaluated	None (0)	No lymphadenopathy (no enlarged lymph node groups)
	Mild (1)	Localized lymphadenopathy (enlargement of one lymph node group)

by imaging) and clinical examination)(7)	Moderate (2)	Generalized lymphadenopathy (enlargement of two non-contiguous lymph node groups)[1]
	Severe (3)	Generalized lymphadenopathy (enlargement of more than two non-contiguous lymph node groups)
Current max. cephalocaudal diameter of the spleen in cm (evaluated by imaging)	None (0)	Maximum cephalocaudal diameter <11 cm
	Mild (1)	Maximum cephalocaudal diameter 11-15 cm
	Moderate (2)	Maximum cephalocaudal diameter 15,1-20 cm
	Severe (3)	Maximum cephalocaudal diameter >20 cm or if information not available because of recent splenectomy to treat splenomegaly (in the last 6 months)
	Please don´t answer and exclude category in the final score calculation	Information not available because of splenectomy to treat splenomegaly in the past (>6 months ago)
**Liver involvement**		
Current status of liver disease	None (0)	No liver disease
	Mild (1)	GGT > 2x normal value(10)
	Moderate (2)	1) GGT > 2x normal value<br>AND<br>2) >6.9 kPa liver stiffness index in Fibro Scan OR spleen > 16cm<br>(Likely portal hypertension)
	Severe (3)	Ascites OR oesophageal varices (Proved portal hypertension)
	Please don´t answer and exclude category in the final score calculation	Liver disease caused by disease independent circumstances (e.g., ascites following alcohol induced liver cirrhosis)

Respiratory involvement		
Number of respiratory tract infections (upper (URTI) and lower respiratory tract infections (LRTI)) in the last 6 months(5)	None (0)	0
	Mild (1)	1 URTI
	Moderate (2)	2-3 URTI or 1 pneumonia or chronic symptoms
	Severe (3)	>3 URTI or >1 pneumonias
Number of antibiotic courses to treat respiratory tract infections (in the last 6 months)	None (0)	No course of antibiotics
	Mild (1)	1 course of antibiotics
	Moderate (2)	2 courses of antibiotics
	Severe (3)	3 or more courses of antibiotics or prophylactic antibiotic treatment to prevent respiratory tract infections
Current diffusion capacity in DLCOc/VA %(7)	None (0)	Normal diffusion capacity (DLCOc/VA >85%
	Mild (1)	Mild impairment of diffusion capacity (DLCOc/VA 70-85%)
	Moderate (2)	Moderate impairment of diffusion capacity (DLCOc/VA 55-69%)
	Severe (3)	Severe impairment of diffusion capacity (DLCOc/VA <55%)
Current MRC Scale value(11),(12)	None (0)	1 (Not troubled by breathlessness except on strenuous exercise)
	Mild (1)	2 (Short of breath when hurrying on a level or when walking up a slight hill)
	Moderate (2)	3 (Walks slower than most people on the level, stops after a mile or so, or stops after 15 minutes walking at own pace)
	Severe (3)	4-5 (4: Stops for breath after walking 100 yards, or after a few minutes on level ground; 5: Too breathless to leave the house, or breathless when dressing/undressing)
Diffuse lung pathology (ground glass opacities and/or consolidation in thorax CT, evaluated by radiologist*)*	None (0)	none
	Mild (1)	<33%
	Moderate (2)	<66%
	Severe (3)	>/= 66%
	None (0)	None

Focal lung pathology (number of perilymphatic (=peribronchovascular) nodules in thorax CT, evaluated by radiologist	Mild (1)	<5
	Moderate (2)	5-10
	Severe (3)	>10
Reticular lung pathology in thorax CT, evaluated by radiologist	None (0)	No Reticulation
	Mild (1)	>0% reticulation without distortion
	Severe (3)	>0% reticulation with distortion
Bronchial wall thickening in thorax CT, evaluated by radiologist (BW=bronchial wall; V=vessel)	None (0)	No bronchial wall thickening
	Mild (1)	BW <0,5 x V
	Moderate (2)	0,5 x V < BW < V
	Severe (3)	BW >/= V
**Skin**		
Current Severity of skin lesions (including fasciitis, vitiligo, aphthous, rosacea-like or psoriasis-like skin lesions)	None (0)	No skin lesions
	Mild (1)	No treatment necessary
	Moderate (2)	Topical treatment necessary
	Severe (3)	Systemic treatment necessary
Current distribution of skin involvement(7)	None (0)	No skin lesions
	Mild (1)	<5% of body surface involved
	Moderate (2)	5-10% of body surface involved
	Severe (3)	>10% of body surface involved
Alopecia: current % of affected scalp	None (0)	0 %
	Mild (1)	<30%
	Moderate (2)	<90%
	Severe (3)	>/=90%
**Gastrointestinal (GI) involvement**		
Current GI involvement (any GI symptoms, especially enteropathy manifesting e.g. with diarrhea)	None (0)	No GIT involvement
	Mild (1)	No treatment necessary
	Moderate (2)	Topical treatment necessary
	Severe (3)	Systemic treatment necessary
Stool frequency and quality within the last 24h(7)	None (0)	Normal stool consistency and under 3 stools
	Mild (1)	>/= 3 to 4 stools or watery stools

	Moderate (2)	>/= 5 to 9 stools
	Severe (3)	>/= 10 stools
**Vasculitis**		
Current Vasculitis/Behcet's disease	None (0)	No vasculitis symptoms
	Mild (1)	Vasculitis suspected in connection with current symptoms
	Severe (3)	Vasculitis diagnosed and currently symptomatic (diagnose not necessarily by biopsy, imaging is also possible)
**Neurological involvement**		
Current neurological impairment (assessment based on neurological examination using the NANO Scale)(7)	None (0)	0 points, no seizures
	Mild (1)	1-2 points and/or 1 or more seizures/year
	Moderate (2)	3-4 points and/or 1 or more seizures/month
	Severe (3)	>/= 5 points and/or 1 or more seizures/week
**Rheumatological involvement**		
Current amount of sIL2 receptor in U/ml(7)	None (0)	<700
	Mild (1)	>/=700
	Moderate (2)	>1200
	Severe (3)	>1700
Current DAS28-CRP-Score value(13),(14)	None (0)	<2.6 (remission)
	Mild (1)	</= 3.2
	Moderate (2)	</= 5.1
	Severe (3)	>5.1
**Non-respiratory infections**		
Number of chronic or transient (viral/bacterial/fungal/parasite) infections (in the last 6 months)	None (0)	No infections
	Mild (1)	1-2 infections
	Moderate (2)	3 infections
	Severe (3)	>3 infections or recurrent infections
Number of chronic infections (in the last 6 months)	None (0)	No chronic infections
	Moderate (2)	1 chronic infection
	Severe (3)	>1 chronic infection
	None (0)	No other infections

Severity of other (viral/bacterial/fungal/parasite) infections (in the last 6 months)	Mild (1)	No treatment necessary
	Moderate (2)	Topical treatment necessary
	Severe (3)	Systemic treatment necessary
Number of antibiotic courses to treat other infections (in the last 6 months)	None (0)	No course of antibiotics
	Mild (1)	1 course of antibiotics
	Moderate (2)	2 courses of antibiotics
	Severe (3)	3 or more courses of antibiotics or prophylactic antibiotic treatment to prevent non-respiratory infections
**Other autoimmune/autoinflammatory conditions (that have not been scored yet)**		
Current other autoimmune/autoinflammatory manifestation, e.g. AI Thyroiditis (or suspicion of)	None (0)	no
	Mild (1)	1 other autoimmune/autoinflammatory organ manifestation
	Moderate (2)	2 other autoimmune/autoinflammatory organ manifestations
	Severe (3)	3 or more other autoimmune/autoinflammatory organ manifestations
Current non-infectious fever	0	No
	3	yes

Table S2: The *NFKB1*-DAS is indicated as a percentage value corresponding to the achieved score points relative to the maximum score value. The maximum possible score value results from the maximum number of points of all parameters with available data. Zero to three points can be achieved for each parameter (0 = not present, 1 = mild, 2 = moderate, 3 = severe). MRC dyspnoea scale used with the permission of the Medical Research Counsil, no changes made, it was integrated in 0-3 points according to the NFKB1-DAS scoring system. .

Table S3: Forty-one individuals carrying heterozygous pathogenic variants in NFKB1 form the study cohort for the development of the NKFB1-DAS. Additionally, 14 patients with a VUS in NFKB1 were identified.

Patient ID	Variant ID	cDNA change	Protein change (*=stop)	Path./VUS	published before
NFKB1-48	NFKB1-variant-1	c.159+1 G>A	p.Ala40Glufs*10 (assumed)	Path.	In ClinVar by 2 Labs
NFKB1-26	NFKB1-variant-2	c.407+1 G>A	p.Ile87Leufs*16 (assumed)	Path.	In ClinVar by 2 Labs
NFKB1-10	NFKB1-variant-3	c.295C>T	p.Gln99*	Path.	PMID: 34473196; PMID: 32278790 [X.I.1]; PMID:29403474 [Patient 7 (13)]; PMID:27923702 [Patient 7]; GRID:001142 [FR005]
NFKB1-40	NFKB1-variant-4	c.469C>T	p.Arg157*	Path.	PMID: 34473196; PMID: 32278790
NFKB1-11	NFKB1-variant-5	c.520_521insCTGA	p.Leu176*	Path.	PMID: 34473196; PMID: 34975878 [Fam.F069:P069]; PMID:32278790 [AC.I.1]
NFKB1-41	NFKB1-variant-6	c.584_585delAG	p.Glu195Alafs*29	Path.	In ClinVar
NFKB1-54	NFKB1-variant-7	c.589delA	p.Ile197Serfs*55	Path.	10.1101/2022.11.22.517350v1 [FR302]

NFKB1-36	NFKB1-variant-8	c.634_656dupGTGGTGCGGCTCATGTTTACAGC	p.Phe220Trpfs*40	Path.	PMID: 34975878 [Fam.F073:P073]
NFKB1-18	NFKB1-variant-8	c.634_656dup	p.Phe220Trpfs*40	Path.	PMID: 34975878
NFKB1-31	NFKB1-variant-8	c.634_656dup	p.Phe220Trpfs*40	Path.	PMID: 34975878
NFKB1-45	NFKB1-variant-9	c.850C>T	p.Arg284*	Path.	PMID: 34473196; PMID: 32278790
NFKB1-24	NFKB1-variant-9	c.850C>T	p.Arg284*	Path.	PMID: 34473196; PMID: 32278790
NFKB1-28	NFKB1-variant-9	c.850C>T	p.Arg284*	Path.	PMID: 34473196; PMID: 32278790
NFKB1-2	NFKB1-variant-10	c.872delA	p.Asn291Metfs*141	Path.	PMID: 34473196; PMID: 32278790 [Fam.AJ:III.3(III.3)]; GRID:001142 [Fam.*:III.3(FR097.2)]
NFKB1-30	NFKB1-variant-10	c.872delA	p.Asn291Metfs*141	Path.	PMID: 34473196; PMID: 34975878 [Fam.F039:P039]; PMID:32278790 [Fam.AJ:III.2(III.2)]

NFKB1-17	NFKB1-variant-10	c.872delA	p.Asn291Metfs*141	Path.	PMID: 34473196; PMID: 32278790 [Fam.AJ:II.2(II.2)]; GRID:001142 [Fam.*:II.2(FR098)]
NFKB1-33	NFKB1-variant-10	c.872delA	p.Asn291Metfs*141	Path.	PMID: 34473196; PMID: 32278790 [Fam.AJ:II.5(II.5)]; GRID:001142 [Fam.+:II.6(FR101)]
NFKB1-7	NFKB1-variant-10	c.872delA	p.Asn291Metfs*141	Path.	PMID: 34473196; PMID: 32278790 [Fam.AJ:III.9(III.9)]; GRID:001142 [Fam.+:III.9(FR100)]
NFKB1-21	NFKB1-variant-10	c.872delA	p.Asn291Metfs*141	Path.	PMID: 34473196; PMID: 34975878 [Fam.F274:P274]; PMID:32278790 [AK.I.1]
NFKB1-34	NFKB1-variant-10	c.872delA	p.Asn291Metfs*141	Path.	PMID: 34473196; PMID: 32278790 [Fam.AJ:III.7(III.7)]; GRID:001142 [Fam.+:III.7(FR103)]
NFKB1-42	NFKB1-variant-11	c.904dupT	p.Ser302Phefs*7	Path.	PMID: 34473196; PMID: 32278790
NFKB1-5	NFKB1-variant-11	c.904dupT	p.Ser302Phefs*7	Path.	PMID: 34473196; PMID: 32278790

NFKB1-38	NFKB1-variant-11	c.904dupT	p.Ser302Phefs*7	Path.	PMID: 34473196; PMID: 32278790
NFKB1-29	NFKB1-variant-11	c.904dupT	p.Ser302Phefs*7	Path.	PMID: 34473196; PMID: 32278790
NFKB1-32	NFKB1-variant-12	c.1066+1G>C	p.Phe310Ilefs*76	Path.	PMID: 34975878 [Fam.F005:P005]; PMID:32278790 [Fam.AU:II.3(II.3)]; PMID:29403474 [Patient 28 (11)]; PMID:27461466 [Fam.199_01:28]
NFKB1-14	NFKB1-variant-13	c.1012delT	p.Ser338Leufs*94	Path.	PMID: 34473196; PMID: 34975878 [Fam.F259:P259]; PMID:32278790 [Fam.AT:III.2(III.2)]; PMID:31803180 [Fam.C:III.1(III.1)]
NFKB1-49	NFKB1-variant-14	c.1071_1074delAGAA	p.Glu358Lysfs*73	Path.	PMID: 34473196; PMID: 32278790 [AW.I.1]
NFKB1-8	NFKB1-variant-20	c.835+2T>G	p.Lys244_Asp279delinsAsn	Path.	PMID: 32278790 [Fam.089:I.1(I.1)]; PMID:26279205 [Fam.089:I.1(I1)]; PMID:29403474 [Fam.089 (5):I.1(I1)]; PMID:27461466 [Fam.089:I.1(24)]
NFKB1-4	NFKB1-variant-20	c.835+2T>G	p.Lys244_Asp279delinsAsn	Path.	PMID: 32278790 [Fam.089:III.2(III.2)]; PMID:26279205 [Fam.089:III.2(III2)]; PMID:29403474 [Fam.089 (5):III.2(III2)]

NFKB1-44	NFKB1-variant-20	c.835+2T>G	p.Lys244_Asp279delinsAsn	Path.	PMID: 34975878 [Fam.F156:P156]; PMID:32278790 [Fam.089:II.2(II.2)]; PMID:26279205 [Fam.089:II.2(II2)]; PMID:29403474 [Fam.089 (5):II.2(II2)]; PMID:27923702 [Patient 5]; PMID:27461466 [Fam.089:II.2(25)]
NFKB1-43	NFKB1-variant-20	c.835+2T>G	p.Lys244_Asp279delinsAsn	Path.	PMID: 26279205; PMID: 34975878 [Fam.F192:P192]; PMID:32278790 [AH.I.1]; PMID:31857261 [Patient 5]; GRID:001142 [FR023]
NFKB1-55	NFKB1-variant-20	c.835+2T>G	p.Lys244_Asp279delinsAsn	Path.	PMID: 32278790 [Fam.089:III.4(III.4)]; PMID:26279205 [Fam.089:III.4(III4)]
NFKB1-47	NFKB1-variant-22	c.856T>A	p.Tyr286Asn	Path.	PMID: 34473196; PMID: 32278790 [BX.I.1]; PMID:36105815 [Y286N]
NFKB1-15	NFKB1-variant-15	c.1361dupA	p.Asn454Lysfs*11	Path.	In ClinVar submitted by one Lab.
NFKB1-39	NFKB1-variant-16	c.1365delT	p.Val456*	Path.	PMID: 32278790 [Fam.BF:II.1(II.1)]; PMID:29403474 [Fam.(13):II.2(Patient 3)]; PMID:27923702 [Fam.191:II.2(Patient 3)]
NFKB1-19	NFKB1-variant-16	c.1365delT	p.Val456*	Path.	PMID: 34975878 [Fam.F160:P160]; PMID:32278790 [Fam.BF:II.2(II.2)]; PMID:29403474 [Fam.(13):II.1(Patient 2)]; PMID:27923702 [Fam.191:II.1(Patient 2)]; GRID:001142 [Fam.#:II.2(FR036)]

NFKB1-6	NFKB1-variant-16	c.1365delT	p.Val456*	Path.	PMID: 27923702; PMID: 32278790 [Fam.BF:III.2(III.2)]; GRID:001142 [Fam.#:III.1(FR113)]
NFKB1-27	NFKB1-variant-17	c.1819delG	p.Ala607Profs*3	Path.	In ClinVar submitted by one Lab.
NFKB1-52	NFKB1-variant-18	c.2455C>T	p.Gln819*	Path.	In ClinVar submitted by one Lab.
NFKB1-51	NFKB1-variant-19	c.2670delG	p.Ala891Glnfs*6	Path.	PMID: 34473196; PMID: 34975878 [Fam.F108:P108]; PMID:33486103 [Fam.1:II.1(patient)]
NFKB1-9	NFKB1-variant-21	c.169C>T	p.Arg57Cys	Path.	PMID: 34473196; PMID: 34975878 [Fam.F413:P413]; PMID:32278790 [Fam.BK:II.1(II.1)]; PMID:36105815 [R57C/1]
NFKB1-22	NFKB1-variant-23	c.641G>A	p.Arg214Gln	VUS	PMID: 34473196; PMID: 34975878 [Fam.F219:P219]; PMID:34975878 [Fam.F219:P219]; PMID:32278790 [BR.I.1]; PMID:32278790 [BR.I.1]; PMID:36105815 [Fam.R214Q:II.2(R214Q/1)]; PMID:36105815 [Fam.R214Q:II.2(R214Q/1)]
NFKB1-20	NFKB1-variant-24	c.734C>T	p.Ala245Val	VUS	PMID: 34473196; PMID: 32278790 [BU.I.1]; PMID:36105815 [A245V]

NFKB1-37	NFKB1-variant-25	c.307A>G	p.Asn103Asp	VUS	PMID:36105815 [N103D]
NFKB1-35	NFKB1-variant-26	c.646A>G	p.Met216Val	VUS	PMID: 34473196; PMID: 34975878 [Fam.F188:P188]; PMID:32278790 [Fam.BS:II.1(II.1)]; PMID:36105815 [Fam.M216V:II.1(M216V/1)]
NFKB1-12	NFKB1-variant-27	c.150A>C	p.Gln50His	VUS	PMID: 34975878 [Fam.F301:P301]
NFKB1-12	NFKB1-variant-28	c.156A>C	p.Lys52Asn	VUS	PMID: 34975878 [Fam.F301:P301]
NFKB1-23	NFKB1-variant-29	c.269A>C	p.Tyr90Ser	VUS	PMID: 34473196; PMID: 34975878 [Fam.F134:P134]; PMID:32278790 [BN.I.1]; PMID:36105815 [Y90S]
NFKB1-16	NFKB1-variant-30	c.736C>A	p.Pro246Thr	VUS	PMID: 34473196; PMID: 32278790 [BV.I.1]; PMID:36105815 [P246T]
NFKB1-46	NFKB1-variant-31	c.2634A>T	p.Arg878Ser	VUS	PMID: 34473196

NFKB1-1	NFKB1-variant-32	c.1845G>T	p.Leu615Phe	VUS	PMID: 34473196; PMID: 35486341 [P017]
NFKB1-13	NFKB1-variant-32	c.1845G>T	p.Leu615Phe	VUS	PMID: 34473196
NFKB1-3	NFKB1-variant-32	c.1845G>T	p.Leu615Phe	VUS	PMID: 34473196
NFKB1-53	NFKB1-variant-33	c.1601G>A	p.Arg534His	VUS	PMID: 34473196
NFKB1-25	NFKB1-variant-34	c.2548C>T	p.Arg850Trp	VUS	PMID: 34473196
NFKB1-50	NFKB1-variant-35	c.1424C>G	p.Ala475Gly	VUS	PMID: 34473196

HET = heterozygous; path. = pathogenic; VUS = variant of unknown significance

Table S4: Thirty-one individuals carrying heterozygous pathogenic variants in *CTLA4* (recruited at the CCI in Freiburg) form the control cohort.

Patient ID	CTLA4 Variant ID	cDNA change	protein change (* = stop)	Variant 'type'
CTLA4-1	#1	c.105C>A	p.Cys35*-	Nonsense
CTLA4-2	#1	c.105C>A	p.Cys35*-	Nonsense
CTLA4-3	#12	c.487del	p.Leu163Serfs*24	Frameshift
CTLA4-4	#1	c.105C>A	p.Cys35*	Nonsense
CTLA4-5	#8	c.356T>G	p.Leu119Arg	Missense
CTLA4-6	#9	c.374dupG	p.Leu126Thrfs*28	Frameshift
CTLA4-7	#15	c.109+1G>T	?	Splice Donor
CTLA4-8	#4	c.214A>C	p.Thr72Pro	Missense
CTLA4-9	#6	c.224G>A	p.Arg75Gln	Missense
CTLA4-10	#13	c.511A>C	p.Ser171Arg	Missense
CTLA4-11	#14	c.531_544del	p.Phe179Cysfs*29	Frameshift
CTLA4-12	#13	c.511A>C	p.Ser171Arg	Missense
CTLA4-13	#15	c.109+1G>T	?	Splice Donor
CTLA4-14	#15	c.109+1G>T	?	Splice Donor
CTLA4-15	#15	c.109+1G>T	?	Splice Donor
CTLA4-16	#2	c.165_190dup	p.Gly64Alafs*17	Frameshift
CTLA4-17	#7	c.326G>A	p.Gly109Glu	Missense
CTLA4-18	#16	c.118G>A	p.Val40Met	Missense
CTLA4-19	#1	c.105C>A	p.Cys35*	Nonsense

CTLA4-20	#5	c.223C>T	p.Arg75Trp	Missense
CTLA4-21	#2	c.165_190dup	p.Gly64Alafs*17	Frameshift
CTLA4-22	#11	c.416A>G	p.Tyr139Cys	Missense
CTLA4-23	#10	c.407C>T	p.Pro136Leu	Missense
CTLA4-24	#7	c.326G>A	p.Gly109Glu	Missense
CTLA4-25	#15	c.109+1G>T	?	Missense
CTLA4-26	#2	c.165_190dup	p.Gly64Alafs*17	Frameshift
CTLA4-27	#5	c.223C>T	p.Arg75Trp	Missense
CTLA4-28	#13	c.511A>C	p.Ser171Arg	Missense
CTLA4-29	#1	c.105C>A	p.Cys35*	Nonsense
CTLA4-30	#7	c.326G>A	p.Gly109Glu	Missense
CTLA4-31	#3	c.209G>A	p.Arg70Gln	Missense

Table S5: Sixteen distinct pathogenic variants could be found in the *CTLA4* control cohort.

CTLA4 Variant ID	cDNA change	protein change (* = stop)	Variant 'type'	AF	published in (PMID)
#1	c.105C>A	p.Cys35*	Nonsense	NA	25329329
#2	c.165_190dup	p.Gly64Alafs*17	Frameshift	NA	34975878
#3	c.209G>A	p.Arg70Gln	Missense	1.86e-6	34111452
#4	c.214A>C	p.Thr72Pro	Missense	NA	37740092
#5	c.223C>T	p.Arg75Trp	Missense	1.24e-6	25329329
#6	c.224G>A	p.Arg75Gln	Missense	8.67e-6	37740092
#7	c.326G>A	p.Gly109Glu	Missense	2.35e-4	37740092
#8	c.356T>G	p.Leu119Arg	Missense	NA	37740092
#9	c.374dupG	p.Leu126Thrfs*28	Frameshift	NA	37406177
#10	c.407C>T	p.Pro136Leu	Missense	NA	37740092
#11	c.416A>G	p.Tyr139Cys	Missense	NA	37740092
#12	c.487del	p.Leu163Serfs*24	Frameshift	NA	37740092
#13	c.511A>C	p.Ser171Arg	Missense	NA	37740092
#14	c.531_544del	p.Phe179Cysfs*29	Frameshift	NA	37740092
#15	c.109+1G>T	?	Splice Donor	NA	37740092
#16	c.118G>A	p.Val40Met	Missense	NA	34975878

AF=allele frequency (from gnomAD accessed on 2026-07-13)

Table S6: Application of the NFKB1-DAS to the study cohort of 41 NFKB1-insufficient patients showed the frequency of clinical manifestations.

Criteria	Score value	Description	Number of patients with available data (out of cohort of 41 included patients)	% of patients with available data (out of cohort of 41 included patients)	Number of Patients scoring in the respective category	% of Patients scoring in the respective category (out of all patients with available data)	Number of Patients scoring in the respective category	% of patients scoring in the respective category (out of all patients with available data)
**Blood**								
**Current IgG level in mg/dl**	None (0)	>/= 700	32	78	6	19%	6	19%
	Mild (1)	400-699			5	16%	26	81%
	Moderate (2)	200-399			1	3%		
	Severe (3)	<200 or IgG replacement			20	62%*		
**Current IgA level in mg/dl**	None (0)	>70	30	73	8	27%	8	27%
	Mild (1)	10-70			10	33%	22	73%
	Severe (3)	<10			12	40%		
**Current IgM level in mg/dl**	None (0)	>40	30	73	6	20%	6	20%
	Mild (1)	31-40			8	27%	24	80%
	Moderate (2)	21-30			3	10%		
	Severe (3)	</=20			13	43%		
	None (0)	>/=1000	39	95	26	67%	26	67%

**Current number of lymphocytes/µl**	Mild (1)	<1000			7	18%	13	33%
	Moderate (2)	<750			2	5%		
	Severe (3)	<500			4	10%		
**Current number of CD4+ T cells/µl**	None (0)	>500	33	80	19	58%	19	58%
	Mild (1)	351-500			6	18%	14	42%
	Moderate (2)	200-350			3	9%		
	Severe (3)	<200			5	15%		
**Current CD8-T cell activation (% of HLA-DR+ CD8+ cells in all CD8+ cells)**	None (0)	< 35%	22	54	14	64%	14	64%
	Mild (1)	<40%			1	4%*	8	36%
	Moderate (2)	<60%			3	14%		
	Severe (3)	≥60%			4	18%		
**Current PD1 expression on CD4+ cells (% of CD4+PD1+ in all CD4+)**	None (0)	<20%	14	34	6	43%	6	43%
	Mild (1)	<30%			1	7%	8	57%
	Moderate (2)	<40%			2	14%		
	Severe (3)	>/= 40%			5	36%		
**Current percentage of switched memory B cells in all B cells (% of CD19/20+,IgM-,CD27+ in all B cells)**	None (0)	>/= 6%	19	46	1	5%	1	5%
	Mild (1)	<6%			3	16%	18	95%
	Moderate (2)	<4%			6	32%		

	Severe (3)	< 2%			9	47%		
**Lymphadenopathy**								
**Current max. cephalocaudal diameter of the spleen in cm (evaluated by imaging)**	None (0)	Maximum cephalocaudal diameter <11cm	17	41	4	24%	4	24%
	Mild (1)	Maximum cephalocaudal diameter 11-15cm			6	35%	13	76%
	Moderate (2)	Maximum cephalocaudal diameter 15,1-20cm			6	35%		
	Severe (3)	Maximum cephalocaudal diameter >20cm or if information not available because of recent splenectomy to treat splenomegaly (in the last 6 months)			1	6%		
	Please don´t answer and exclude category in the final	Information not available because of splenectomy to treat splenomegaly in the past (>6 months ago)			/	/	/	/

	score calculation							
**Liver involvement**								
**Current status of liver disease**	None (0)	No liver disease	28	68	19	68%	19	68%
	Mild (1)	GGT > 2x normal value			4	14%	9	32%
	Moderate (2)	1) GGT > 2x normal value AND 2) >6.9 kPa liver stiffness index in Fibro Scan OR spleen > 16cm (Likely portal hypertension)			3	11%		
	Severe (3)	Ascites OR oesophageal varices (Proved portal hypertension)			2	7%		
	Please don´t answer and exclude category in the final	Liver disease caused by disease independent circumstances (e.g., ascites following alcohol induced liver cirrhosis)			/	/	/	/

	score calculation							
**Respiratory involvement**								
**Number of respiratory tract infections (URTI+LRTI) in the last 6 months**	None (0)	0	40	98	16	40%	16	40%
	Mild (1)	1 URTI			6	15%	24	60%
	Moderate (2)	2-3 URTI or 1 pneumonia or chronic symptoms			13	32%*		
	Severe (3)	>3 URTI or >1 pneumonias			5	13%		
**Number of antibiotic courses to treat respiratory tract infections (in the last 6 months)**	None (0)	No course of antibiotics	40	98	27	67%*	27	67%*
	Mild (1)	1 course of antibiotics			6	15%	13	33%
	Moderate (2)	2 courses of antibiotics			4	10%		
	Severe (3)	3 or more courses of antibiotics or prophylactic antibiotic treatment to prevent respiratory tract infections			3	8%		
**Current MRC Scale value**	None (0)	1 (Not troubled by breathlessness except on strenuous exercise)	35	85	24	68%*	24	68%*
	Mild (1)	2 (Short of breath when hurrying on a level or when walking up a slight hill)			8	23%	11	32%*

	Moderate (2)	3 (Walks slower than most people on the level, stops after a mile or so, or stops after 15 minutes walking at own pace)			2	6%		
	Severe (3)	4-5 (4: Stops for breath after walking 100 yards, or after a few minutes on level ground; 5: Too breathless to leave the house, or breathless when dressing/undressing)			1	3%		
**Skin**								
**Current Severity of skin lesions (including fasciitis, vitiligo, aphthous, rosacea-like or psoriasis-like skin lesions)**	None (0)	No skin lesions	40	98	24	60%	24	60%
	Mild (1)	No treatment necessary			6	15%	16	40%
	Moderate (2)	Topical treatment necessary			7	17%*		
	Severe (3)	Systemic treatment necessary			3	8%		
**Current distribution of skin involvement**	None (0)	No skin lesions	39	95	24	62%	24	62%
	Mild (1)	<5% of body surface involved			8	20%*	15	38%
	Moderate (2)	5-10% of body surface involved			6	15%		

	Severe (3)	>10% of body surface involved			1	3%		
**Gastrointestinal involvement**								
**Current GIT involvement**	None (0)	No GIT involvement	41	100	26	64%*	26	64%*
	Mild (1)	No treatment necessary			3	7%	15	36%*
	Moderate (2)	Topical treatment necessary			5	12%		
	Severe (3)	Systemic treatment necessary			7	17%		
**Stool frequency and quality within the last 24h**	None (0)	Normal stool consistency and under 3 stools	40	98	29	72%*	29	72%*
	Mild (1)	>/= 3 to 4 stools or watery stools			4	10%	11	28%
	Moderate (2)	>/= 5 to 9 stools			5	13%		
	Severe (3)	>/= 10 stools			2	5%		
**Rheumatological involvement**								
**Current amount of sIL2 receptor in U/ml**	None (0)	<700	28	68	15	54%	15	54%
	Mild (1)	>/=700			7	25%	13	46%
	Moderate (2)	>1200			2	7%		
	Severe (3)	>1700			4	14%		
**Non-respiratory infections**								
	None (0)	No infections	35	85	22	63%	22	63%

**Number of chronic or transient (viral/bacterial/fungal/parasite) infections (in the last 6 months)**	Mild (1)	1-2 infections			7	20%	13	37%
	Moderate (2)	3 infections			1	3%		
	Severe (3)	>3 infections or recurrent infections			5	14%		
**Severity of other (viral/bacterial/fungal/parasite) infections (in the last 6 months)**	None (0)	No other infections	37	90	22	59%	22	59%
	Mild (1)	No treatment necessary			1	3%	15	41%
	Moderate (2)	Topical treatment necessary			4	11%		
	Severe (3)	Systemic treatment necessary			10	27%		
**Other autoimmune/autoinflammatory conditions (that have not been scored yet)**								
**Current other autoimmune/autoinflammatory manifestation, e.g. AI Thyroiditis (or suspicion of)**	None (0)	no	41	100	19	46%	19	46%
	Mild (1)	1 other autoimmune/autoinflammatory organ manifestation			15	37%	22	54%
	Moderate (2)	2 other autoimmune/autoinflammatory organ manifestations			3	7%		
	Severe (3)	3 or more other autoimmune/autoinflammatory organ manifestations			4	10%		

Table S6: Unless otherwise stated, the percentages given refer to the number of patients with available data. Some percentage values were rounded and adapted to a total value of 100% (marked with *). MRC dyspnoea scale used with the permission of the Medical Research Counsil, no changes made, it was integrated in the 0-3 point scoring system of the NFKB1-DAS.

Table S7: Thirty-one CTLA4-deficient patients form the control cohort.

	**Patients with variants in *CTLA4***
**Total number of patients**	31
**Male (number/%)**	12/39
**Female (number/%)**	19/61
**Mean age at first survey date (years)**	41
**Median age at first survey date (years)**	39
**Minimal age at first survey date (years)**	20
**Maximum age at first survey date (years)**	71
**Deceased during the study period (number)**	0

FigS1: The NFKB1-DAS was developed in four consecutive steps

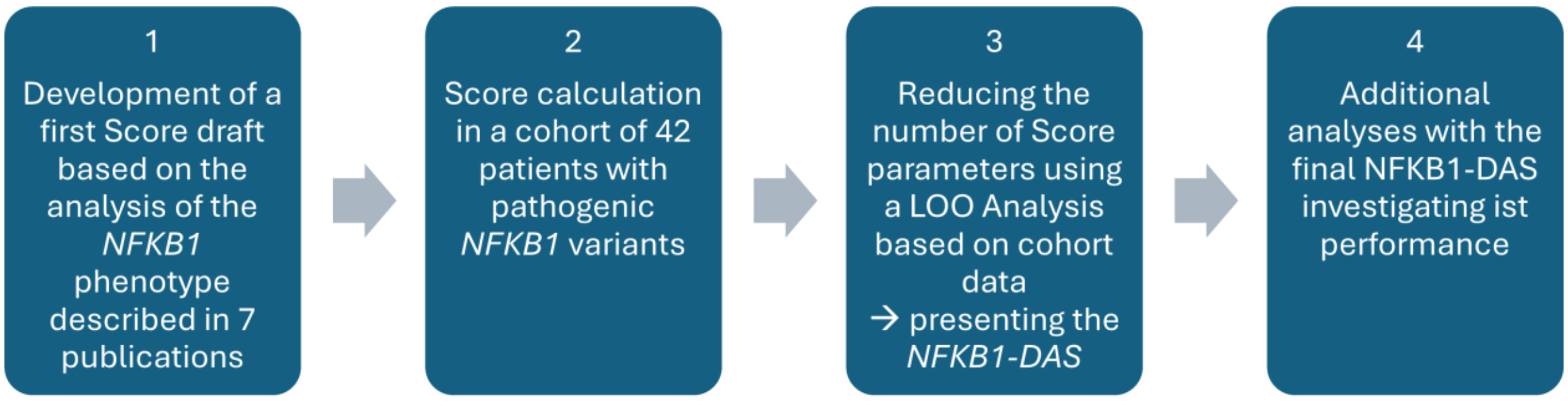

FigS1: Based on the published *NFKB1* phenotype, we developed an initial score draft (1), which was then calculated in a multicenter cohort of 41 patients with pathogenic variants in *NFKB1* (2). Based on these data, we performed a leave one out (LOO) analysis, which enabled us to halve the number of score parameters, leading to the final *NFKB1*-DAS (3). To get a first impression of the performance of the *NFKB1*-DAS in familiar diseases it was calculated in 31 patients with *CTLA4* deficiency and following the hypothesis that it could serve as a diagnostic tool in 14 patients with VUS in *NFKB1*.

FigS2: A representative sample shows the FACS gating strategy.

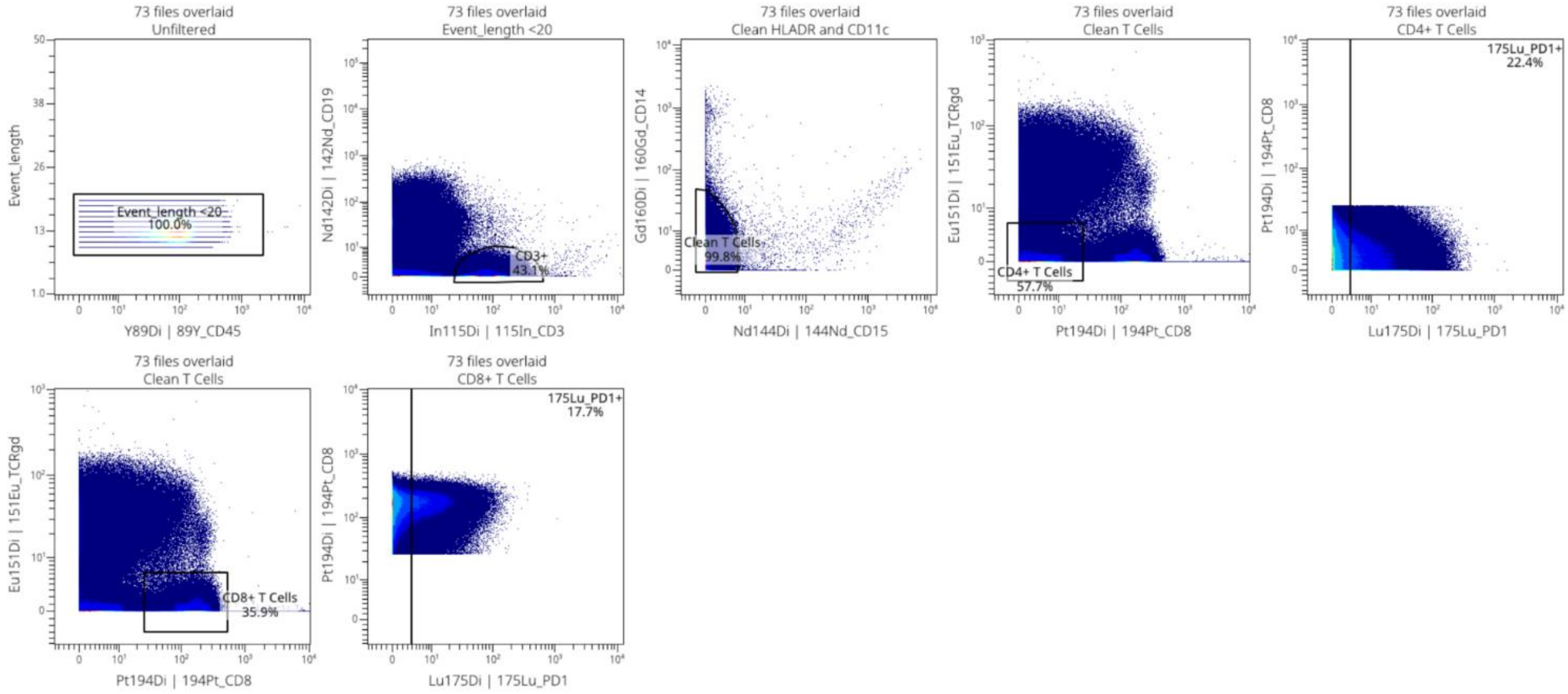

FigS2: CD4^{+}TCRgd^{-} and CD8^{+}TCRgd^{-} T cells (defined as CD3^{+}CD19^{-}, HLA-DR$^{-/+}$CD11c$^{-/+}$, CD14^{-}CD15^{-} cells), were manually gated into PD-1^{-} and PD-1^{+} populations. All anti-human antibodies pre-conjugated to metal isotopes were obtained from Standard BioTools (San Francisco, USA). All remaining antibodies were obtained from the indicated companies as purified antibodies and in-house conjugation was done using the MaxPar X8 labeling kit (Standard BioTools, USA). Sample processing, cell staining and acquisition was done as previously described.(15) OMIQ.ai cloud-based cytometry analysis software was used for de-barcoding of individual samples, manual gating of singlets and removal of normalization beads and dead cells. Per-channel intensity ranges were aligned between batches using a reference sample - a replicate acquired across all batches, and a proprietary script, based on BatchAdjust function, to compute scaling factors at the event percentiles of choice on per-channel basis (16). Channel intensities were transformed using the inverse hyperbolic sine function (asinh) with the cofactor of 5 and two populations of interest, CD4^{+}TCRgd^{-} and CD8^{+}TCRgd^{-} T cells (defined as CD3^{+}CD19^{-}, HLA-DR$^{-/+}$CD11c$^{-/+}$, CD14^{-}CD15^{-} cells), were manually gated into PD-1^{-} and PD-1^{+} populations.

File S1: Questionnaire used for data collection in adults

NFKB1 Fragebogen – Auszufüllen durch Arzt/Ärztin

**Daten zum Patient/zur Patientin**		**Daten zum Arzt/zur Ärztin/zum Befragenden**	
Datum		Name	
Name		Tel.	
Geburtsdatum		E-Mail	

Vereinbarter Wiedervorstellungstermin (wenn möglich nach 6 Monaten): ______________

**Untersuchungen**[1]	**Entweder:** Ergebnis im Arztbrief dokumentiert	**Oder:** Befund angehängt	**Befund liegt nicht vor**
**Körperliche Untersuchung**<br>(mit Bestimmung der Zahl der vergrößerten abgrenzbaren Lymphknotengruppen)	☐	Bzw. Anzahl hier angeben: _________	☐
**Lungenfunktionsuntersuchung**<br>(mit Bestimmung der Diffusionskapazität)	☐	☐	☐
**Sonographie des Abdomens**<br>(mit Bestimmung der Milz- und Lebergröße sowie Untersuchung auf vergrößerte Lymphknoten)	☐	☐	☐
**CT der Lunge**: falls erfolgt Befund und Bilder bitte verschlüsselt übermitteln			☐
**Laboruntersuchung:**<br>- Kleines Blutbild<br>- Differenzialblutbild, B-, T- und Lymphozytenpanel mit<br>• Lymphozyten/µl<br>• Neutrophile Granulozyten (10^3/µl)<br>• B-Gedächtniszellen (CD19/20+IgD-CD27+ bzw. CD19/20+IgM-CD27+)<br>• CD4-Zellen/µl<br>• Naive T-Zellen (% von CD4+CD45RA+)<br>• Aktivierte CD4+ T-Zellen (% von HLA-DR+ CD4+ in CD4+)<br>• Aktivierte CD8+ T-Zellen (% von HLA-DR+ CD8+ in CD8+)<br>• PD1-Expression bei CD4+ T-Zellen (% von CD4+PD1+ in CD4+)<br>• PD1-Expression bei CD8+ T-Zellen (% von CD8+PD1+ in CD8+)<br>- Kreatinin<br>- Haptoglobin<br>- LDH<br>- Ferritin<br>- Beta2-Mikroglobulin<br>- GPT, gammaGT<br>- CRP<br>- IgG, IgA, IgM<br>- Löslicher Interleukin 2 Rezeptor (sIL2)<br>- Calprotectin im Stuhl<br>- Bei Gelenkbeschwerden<br>• Rheumafaktor<br>• CCP-Antikörper<br>- Falls aktuell keine Ig-Substitution erfolgt:<br>• IgG1, IgG2<br>• Pneumokokken-Antikörpertiter (falls Impfung erfolgt/durchgemachte Infektion)<br>- Falls noch nicht erfolgt/Wiederholung indiziert: ANA, ANCA<br>- Falls indiziert oder positiver Test vorliegend: Coombs-Test	☐	☐	☐

[1] Abdomensonographie und Lungenfunktionsuntersuchung sollen mind. Jährlich erfolgen. B- und T-Panel sowie die PD1-Expression von CD4+ und CD8+ T-Zellen sollen pro Patient im Erhebungszeitraum mind. 2x bestimmt werden, 1x ist ausreichend, falls Befunde aus der Vergangenheit vorliegen.

1

0	**Aktueller Karnofski-Score-Wert** *Score not printed in this publication due to copyright issues. It was originally published in: Karnofsky DA and Burchenal JH. The clinical evaluation of chemotherapeutic agents in cancer. MacLeod CM (ed.) Evaluation of chemotherapeutic agents. New York: Columbia University Press, 1949, pp.191–205.*		

1	**Maligne Erkrankung**	Nein	☐
		Ja, in Komplettremission	☐
		Ja, in Progress	☐

2	**Anzahl respiratorischer Infekte in den letzten 6 Monaten**	Anzahl: ____________
		Davon Pneumonien: __________
	**Bei wie vielen dieser respiratorischen Infekte wurde ein Antibiotikum eingesetzt?**	Anzahl:
	**Aktueller MRC-Score-Wert**	
	Score not printed in this publication due to copyright issues	

3	**Wie viele Antibiotika-Einsätze gab es insgesamt in den letzten 6 Monaten (jegliche Indikation)?**	Anzahl:
	Indikationen: ____	

4	**Andere (virale/bakterielle/parasitäre/Pilz-) Infektionen in den letzten 6 Monaten?**		Nein	☐
			Ja	☐
	Falls ja:			
	**Anzahl an Infektionen in den letzten 6 Monaten**			Anzahl: __________
	Beschreibung	Vorübergehend		☐
		Rezidivierend		☐
		chronisch		☐
	Schweregrad	Keine Behandlung nötig, Lebensqualität nicht eingeschränkt		☐
		Lokale Therapie		☐
		Systemische Therapie		☐

5	**Verdacht auf eine noduläre regenerative Hyperplasie der Leber (NRH)**		Nein	☐
			Ja	☐
	Falls ja: Befunde von Biopsien, Fibroscan-Untersuchungen, Duplex-Sonographie-Untersuchungen und Endoskopien soweit vorliegend bitte verschlüsselt übermitteln			
	Beschreibung der aktuellen Krankheitsaktivität	____		

6	**Alopezie**	Nein	☐
		Ja	☐
	Falls ja:		
	Aktuell betroffene Kopfhaut in %		% angeben: __________
	**Hautbeteiligung**	Nein	☐
		Ja	☐
	Falls ja:		
	Schweregrad	Keine Behandlung	☐
		Lokale Therapie	☐

2

7	**Verdacht auf Vaskulitis oder M. Behcet**	Nein	☐
		Ja	☐
	Falls ja:		
	Systemische Symptomatik (z.B. Gewichtsabnahme, Fieber)	____________________	
	Organspezifische Symptomatik	____________________	
	Ergebnisse von Biopsien (Befunde bitte verschlüsselt übermitteln	____________________	
	**Andere kardiovaskuläre Erkrankung**	Nein	☐
		Ja	☐
	Falls ja:		
	Diagnosen:	____________________	
8	**Gastrointestinale Beteiligung**	Nein	☐
		Ja	☐
	Falls ja:		
	Schweregrad	Keine Behandlung	☐
		Systemische Therapie	☐
		Lokale Therapie	☐
	Wässriger Stuhlgang in den letzten 24h	Nein	☐
		Ja	☐
	Häufigkeit des Stuhlgangs in den letzten 24h		*Anzahl:* ________
9	**Aktivität/Schwere von Gelenkbeschwerden** (zur Berechnung des DAS28-CRP-Scores) :		
	Score not printed in this publication due to copyright issues		
10	**Hat der Patient aktuell nicht-infektiöses Fieber**	Nein	☐
		Ja	☐
	**Andere Autoimmunerkrankungen/hyperinflammatorische Prozesse**	Nein	☐
		Ja	☐
	Falls ja:		
	Anzahl solcher Organmanifestationen		Anzahl: ________
	Bitte Diagnosen hier auflisten:	____________________	
11	**Hatte der Patient epileptische Anfälle?**	Nein	☐
		Ja	☐
	Falls ja:		
	Anzahl der epileptischen Anfälle		Anzahl: ________
	pro	Woche	☐
		Monat	☐
		Jahr	☐
	Erster Anfall (Monat/Jahr):		____/______
	Letzter Anfall (Monat/Jahr):		____/______ Oder:

		☐ nur ein Anfall
**Weist der Patient Einschränkungen in den folgenden Bereichen auf?:** Gang, Stärke, Ataxie, Sinneswahrnehmungen, Gesichtsfeld, mimische Muskulatur, Sprache, Bewusstsein, Verhalten	Nein	☐
	Ja	☐

Falls ja, bitte beantworten Sie die folgenden näheren Fragen zur Berechnung des NANO-Scale-Wertes:
Score not printed in this publication due to copyright issues

12 **Aktuelle Medikation**

Medikament	Dosierung	Start	Grund	Therapieerfolg: welche Symptome haben sich gebessert? Welche nicht?

Nebenwirkungen treten bei den folgenden Medikamenten auf:

Medikament	Nebenwirkung

13		
**Hat der Patient/die Patientin in der Vergangenheit (weitere) immunmodifizierende Medikamente erhalten?**	Nein	☐
	Ja	☐

Falls ja:

Medikation (mit Dosierung)	Start	Ende	Grund	Therapieerfolg: welche Symptome haben sich gebessert? Welche nicht?

4

Nebenwirkungen traten bei den folgenden immunmodifizierenden Medikamenten auf:

Medikation	Nebenwirkung

14	**Kommentar:**

File S2: Questionnaire (children) used for data collection

NFKB1 Fragebogen – Auszufüllen durch Arzt/Ärztin

Bitte alle grau markierten Felder bearbeiten

**Daten zum Patient/zur Patientin**		**Daten zum Arzt/zur Ärztin/zum Befragenden**	
Datum		Name	
Name		Tel.	
Geburtsdatum		E-Mail	

Vereinbarter Wiedervorstellungstermin (wenn möglich nach 6 Monaten): _____________

**Untersuchungen**[1]	**Entweder:** **Ergebnis im Arztbrief dokumentiert**	**Oder:** **Befund angehängt**	**Befund liegt nicht vor**
**Körperliche Untersuchung** (mit Bestimmung der Zahl der vergrößerten abgrenzbaren Lymphknotengruppen)	☐	Bzw. Anzahl hier angeben: ________	☐
**Lungenfunktionsuntersuchung** (mit Bestimmung der Diffusionskapazität)	☐	☐	☐
**Sonographie des Abdomens** (mit Bestimmung der Milz- und Lebergröße sowie Untersuchung auf vergrößerte Lymphknoten)	☐	☐	☐
**CT der Lunge**: falls erfolgt Befund und Bilder bitte verschlüsselt übermitteln			☐
**Laboruntersuchung:**<br>- Kleines Blutbild<br>- Differenzialblutbild, B-, T- und Lymphozytenpanel mit<br>• Lymphozyten/µl<br>• Neutrophile Granulozyten (10^3/µl)<br>• B-Gedächtniszellen (CD19/20+IgD-CD27+ bzw. CD19/20+IgM-CD27+)<br>• CD4-Zellen/µl<br>• Naive T-Zellen (% von CD4+CD45RA+)<br>• Aktivierte CD4+ T-Zellen (% von HLA-DR+ CD4+ in CD4+)<br>• Aktivierte CD8+ T-Zellen (% von HLA-DR+ CD8+ in CD8+)<br>• PD1-Expression bei CD4+ T-Zellen (% von CD4+PD1+ in CD4+)<br>• PD1-Expression bei CD8+ T-Zellen (% von CD8+PD1+ in CD8+)<br>- Kreatinin<br>- Haptoglobin<br>- LDH<br>- Ferritin<br>- Beta2-Mikroglobulin<br>- GPT, gammaGT<br>- CRP<br>- IgG, IgA, IgM<br>- Löslicher Interleukin 2 Rezeptor (sIL2)<br>- Calprotectin im Stuhl<br>- Bei Gelenkbeschwerden<br>• Rheumafaktor<br>• CCP-Antikörper<br>- Falls aktuell keine Ig-Substitution erfolgt:<br>• IgG1, IgG2<br>• Pneumokokken-Antikörpertiter (falls Impfung erfolgt/durchgemachte Infektion)<br>- Falls noch nicht erfolgt/Wiederholung indiziert: ANA, ANCA<br>- Falls indiziert oder positiver Test vorliegend: Coombs-Test	☐	☐	☐

[1] Abdomensonographie und Lungenfunktionsuntersuchung sollen mind. Jährlich erfolgen. B- und T-Panel sowie die PD1-Expression von CD4+ und CD8+ T-Zellen sollen pro Patient im Erhebungszeitraum mind. 2x bestimmt werden, 1x ist ausreichend, falls Befunde aus der Vergangenheit vorliegen.

1

0		Aktueller Karnofsky-Score-Wert (bei Patient*innen ab 16J nutzen)	Aktueller Lansky-Score-Wert (bei Patient*innen <16J nutzen)	

Score not printed in this publication due to copyright issues. The Karnofsky Score was originally published in: Karnofsky DA and Burchenal JH. The clinical evaluation of chemotherapeutic agents in cancer. MacLeod CM (ed.) Evaluation of chemotherapeutic agents. New York: Columbia University Press, 1949, pp.191–205. The Lansky Score was originally published in Lansky, L.L., List, M.A., Lansky, S.B., Cohen, M.E. and Sinks, L.F. (1985) "Toward the development of a play performance scale for children (PPSC)," Cancer, 56(7 Suppl), pp. 1837–1840. Available at: https://doi.org/10.1002/1097-0142(19851001)56:7+%3C1837::aid-cncr2820561324%3E3.0.co;2-z

1	**Maligne Erkrankung**	Nein	☐
		Ja, in Komplettremission	☐
		Ja, in Progress	☐

2	**Anzahl respiratorischer Infekte in den letzten 6 Monaten**	Anzahl: ______
		Davon Pneumonien: ______
	**Bei wie vielen dieser respiratorischen Infekte wurde ein Antibiotikum eingesetzt?**	Anzahl:
	**Aktueller MRC-Score-Wert**	
	Score not printed in this publication due to copyright issues	
3	**Wie viele Antibiotika-Einsätze gab es insgesamt in den letzten 6 Monaten (jegliche Indikation)?**	Anzahl:
	Indikationen: ______________________________	

4	**Andere (virale/bakterielle/parasitäre/Pilz-) Infektionen in den letzten 6 Monaten?**	Nein	☐
		Ja	☐

Falls ja:

**Anzahl an Infektionen in den letzten 6 Monaten**		Anzahl: ______
Beschreibung	Vorübergehend	☐
	Rezidivierend	☐
	chronisch	☐
Schweregrad	Keine Behandlung nötig, Lebensqualität nicht eingeschränkt	☐
	Lokale Therapie	☐
	Systemische Therapie	☐

5	**Verdacht auf eine noduläre regenerative Hyperplasie der Leber (NRH)**	Nein	☐
		Ja	☐

Falls ja: Befunde von Biopsien, Fibroscan-Untersuchungen, Duplex-Sonographie-Untersuchungen und Endoskopien soweit vorliegend bitte verschlüsselt übermitteln

Beschreibung der aktuellen Krankheitsaktivität	______________________________

6	**Alopezie**	Nein	☐
		Ja	☐

Falls ja:

Aktuell betroffene Kopfhaut in %	% angeben: ______

2

	**Hautbeteiligung**	Nein	☐
		Ja	☐
	Falls ja:		
	Schweregrad	Keine Behandlung	☐
		Lokale Therapie	☐
		Systemische Therapie	☐
	Betroffener Anteil der Körperoberfläche in %		% angeben: ________
7	**Verdacht auf Vaskulitis oder M. Behcet**	Nein	☐
		Ja	☐
	Falls ja:		
	Systemische Symptomatik (z.B. Gewichtsabnahme, Fieber)	________________________________	
	Organspezifische Symptomatik	________________________________	
	Ergebnisse von Biopsien	________________________________	
	**Andere kardiovaskuläre Erkrankung**	Nein	☐
		Ja	☐
	Falls ja:		
	Diagnosen:	________________________________	
8	**Gastrointestinale Beteiligung**	Nein	☐
		Ja	☐
	Falls ja:		
	Schweregrad	Keine Behandlung	☐
		Systemische Therapie	☐
		Lokale Therapie	☐
	Wässriger Stuhlgang in den letzten 24h	Nein	☐
		Ja	☐
	Häufigkeit des Stuhlgangs in den letzten 24h		*Anzahl:* ________
9	**Aktivität/Schwere von Gelenkbeschwerden** (zur Berechnung des DAS28-CRP-Scores) :<br>*Score not printed in this publication due to copyright issues*		
10	**Hat der Patient aktuell nicht-infektiöses Fieber**	Nein	☐
		Ja	☐
	**Andere Autoimmunerkrankungen/hyperinflammatorische Prozesse**	Nein	☐
		Ja	☐
	Falls ja:		
	Anzahl solcher Organmanifestationen		*Anzahl:* ________
	Bitte Diagnosen hier auflisten:	________________________________	
11	**Hatte der Patient epileptische Anfälle?**	Nein	☐
		Ja	☐

3

Falls ja:		
Anzahl der epileptischen Anfälle		Anzahl: ___________
pro	Woche	☐
	Monat	☐
	Jahr	☐
Erster Anfall (Monat/Jahr):		____/______
Letzter Anfall (Monat/Jahr):		____/______ Oder: ☐ nur ein Anfall
**Weist der Patient Einschränkungen in den folgenden Bereichen auf?:** Gang, Stärke, Ataxie, Sinneswahrnehmungen, Gesichtsfeld, mimische Muskulatur, Sprache, Bewusstsein, Verhalten	Nein	☐
	Ja	☐
Falls ja, bitte beantworten Sie die folgenden näheren Fragen zur Berechnung des NANO-Scale-Wertes: *Score not printed in this publication due to copyright issues*		

12 **Aktuelle Medikation**

Medikament	Dosierung	Start	Grund	Therapieerfolg: welche Symptome haben sich gebessert? Welche nicht?

Nebenwirkungen treten bei den folgenden Medikamenten auf:

Medikament	Nebenwirkung

13	**Hat der Patient/die Patientin in der Vergangenheit (weitere) immunmodifizierende Medikamente erhalten?**	Nein	☐
		Ja	☐
	Falls ja:		

Medikation (mit Dosierung)	Start	Ende	Grund	Therapieerfolg: welche Symptome haben sich gebessert? Welche nicht?

Nebenwirkungen traten bei den folgenden immunmodifizierenden Medikamenten auf:

Medikation	Nebenwirkung

14	**Kommentar:**

File S3: Questionnaire for the calculation of the final NFKB1-DAS

NFKB1-DAS Questionnaire

Patient name: ____________________ Patient birth date: _________ Date of evaluation: _____________

**EXAMINATION**	
**Abdomen sonography**	
Maximum cephalocaudal diameter of the spleen in cm OR splenectomy because of splenomegaly?	
Fibro Scan (liver stiffness in kPa)	
Ascites or esophageal varices?	
**Laboratory tests**	
Lymphocytes/µl	
Current percentage of switched memory B cells in all B cells (% of CD19/20+,IgM-,CD27+ in all B cells)	
CD4 T cells /µl	
Activated CD8+ T cells (% of HLA-DR+ CD8+ in CD8+)	
PD1 expression in CD4+ T cells (% of CD4+PD1+ in CD4+)	
IgG in mg/dl	
IgA in mg/dl	
IgM in mg/dl	
sIL2 receptor in U/ml	
GGT	

1	**Number of respiratory tract infections**	No. URTI: ____________ No. pneumonias: ____________
	**How many of these infections were treated with antibiotics?**	No.
	**MRC Dyspnoea Scale** (used with the permission of the Medical Research Counsil, no changes made)	
	Grade 1: Are you ever troubled by breathlessness except on strenuous exertion?	☐
	Grade 2: (If yes) Are you short of breath when hurrying on the level or walking up a slight hill?	☐
	Grade 3: (If yes) Do you have to walk slower than most people on the level? Do you have to stop after a mile or so (or after ¼ hour) on the level at your own pace?	☐
	Grade 4: (If yes to either) Do you have to stop for breath after walking about 100 yds. (or after a few minutes) on the level?	☐
	Grade 5: (If yes) Are you too breathless to leave the house, or breathless after undressing?	☐

2	**Number of other viral/bacterial/parasitic/fungal infections**		No. ___________
	Description	temporary	☐
		recurrent	☐
	Severity	No treatment necessary	☐
		Local therapy	☐
		Systemic therapy	☐
3	**Gastrointestinal involvement**	No involvement/ no treatment necessary	☐
		Local therapy	☐
		Systemic therapy	☐
	Watery stool in the last 24 hours	No	☐
		Yes	☐
	Frequency of stool in the last 24 hours		No. ___________
4	**Liver involvement**	No	☐
	Ascites or esophageal varices?	yes	☐
5	**Skin involvement**	No/not therapy	☐
		Local therapy	☐
		Systemic therapy	☐
	% of affected skin (*Hand: 1%, Arm: 9%, Leg: 18%, genital area: 1%, torso: anterior und posterior 18% each, Head and neck: 9%*)	% number	___________
6	**Other autoimmune/autoinflammatory manifestations**		No. __________
	List diagnoses here:	____	

NFKB1-DAS and Calculation procedure developed by Thoma et al. (AG Grimbacher CCI Freiburg).

File S4: Final NFKB1-DAS and calculation procedure

NFKB1-DAS

Criteria	Score value	Description
Blood		
Current IgG level in mg/dl	None (0)	>/= 700
	Mild (1)	400-699
	Moderate (2)	200-399
	Severe (3)	<200 or IgG replacement
Current IgA level in mg/dl	None (0)	>70
	Mild (1)	10-70
	Severe (3)	<10
Current IgM level in mg/dl	None (0)	>40
	Mild (1)	31-40
	Moderate (2)	21-30
	Severe (3)	</=20
Current number of lymphocytes/µl	None (0)	>/=1000
	Mild (1)	<1000
	Moderate (2)	<750
	Severe (3)	<500
Current number of CD4+ T cells/µl	None (0)	>500
	Mild (1)	351-500
	Moderate (2)	200-350
	Severe (3)	<200
Current CD8-T cell activation (% of HLA-DR+ CD8+ cells in all CD8+ cells)	None (0)	< 35%
	Mild (1)	<40%
	Moderate (2)	<60%
	Severe (3)	≥60%
Current PD1 expression on CD4+ cells (% of CD4+PD1+ in all CD4+)	None (0)	<20%
	Mild (1)	<30%
	Moderate (2)	<40%
	Severe (3)	>/= 40%
Current percentage of switched memory B cells in all B cells (% of CD19/20+,IgM-,CD27+ in all B cells)	None (0)	>/= 6%
	Mild (1)	<6%
	Moderate (2)	<4%
	Severe (3)	< 2%

Lymphadenopathy		
Current max. cephalocaudal diameter of the spleen in cm (evaluated by imaging)	None (0)	Maximum cephalocaudal diameter <11cm
	Mild (1)	Maximum cephalocaudal diameter 11-15cm
	Moderate (2)	Maximum cephalocaudal diameter 15,1-20cm
	Severe (3)	Maximum cephalocaudal diameter >20cm or if information not available because of recent splenectomy to treat splenomegaly (in the last 6 months)
	Please don´t answer and exclude category in the final score calculation	Information not available because of splenectomy to treat splenomegaly in the past (>6 months ago)
Liver involvement		
Current status of liver disease	None (0)	No liver disease
	Mild (1)	GGT > 2x normal value
	Moderate (2)	1) GGT > 2x normal value AND<br>2) >6.9 kPa liver stiffness index in Fibro Scan OR spleen > 16cm<br>(Likely portal hypertension)
	Severe (3)	Ascites OR oesophageal varices (Proved portal hypertension)
	Please don´t answer and exclude category in the final score calculation	Liver disease caused by disease independent circumstances (e.g., ascites following alcohol induced liver cirrhosis)

Respiratory involvement		
Number of respiratory tract infections (URTI+LRTI) in the last 6 months	None (0)	0
	Mild (1)	1 URTI
	Moderate (2)	2-3 URTI or 1 pneumonia or chronic symptoms
	Severe (3)	>3 URTI or >1 pneumonias
Number of antibiotic courses to treat respiratory tract infections (in the last 6 months)	None (0)	No course of antibiotics
	Mild (1)	1 course of antibiotics
	Moderate (2)	2 courses of antibiotics
	Severe (3)	3 or more courses of antibiotics or prophylactic antibiotic treatment to prevent respiratory tract infections
Current MRC Scale value *MRC dyspnoea scale used with the permission of the Medical Research Counsil, no changes made, it was integrated in the 0-3 point scoring system of the NFKB1-DAS*	None (0)	1 (Not troubled by breathlessness except on strenuous exercise)
	Mild (1)	2 (Short of breath when hurrying on a level or when walking up a slight hill)
	Moderate (2)	3 (Walks slower than most people on the level, stops after a mile or so, or stops after 15 minutes walking at own pace)
	Severe (3)	4-5 (4: Stops for breath after walking 100 yards, or after a few minutes on level ground; 5: Too breathless to leave the house, or breathless when dressing/undressing)
Skin		
Current Severity of skin lesions (including fasciitis, vitiligo, aphthous, rosacea-like or psoriasis-like skin lesions)	None (0)	No skin lesions
	Mild (1)	No treatment necessary
	Moderate (2)	Topical treatment necessary
	Severe (3)	Systemic treatment necessary
Current distribution of skin involvement	None (0)	No skin lesions
	Mild (1)	<5% of body surface involved

	Moderate (2)	5-10% of body surface involved
	Severe (3)	>10% of body surface involved
Gastrointestinal involvement		
Current GIT involvement	None (0)	No GIT involvement
	Mild (1)	No treatment necessary
	Moderate (2)	Topical treatment necessary
	Severe (3)	Systemic treatment necessary
Stool frequency and quality within the last 24h	None (0)	Normal stool consistency and under 3 stools
	Mild (1)	>/= 3 to 4 stools or watery stools
	Moderate (2)	>/= 5 to 9 stools
	Severe (3)	>/= 10 stools
Rheumatological involvement		
Current amount of sIL2 receptor in U/ml	None (0)	<700
	Mild (1)	>/=700
	Moderate (2)	>1200
	Severe (3)	>1700
Non-respiratory infections		
Number of chronic or transient (viral/bacterial/fungal/parasite) infections (in the last 6 months)	None (0)	No infections
	Mild (1)	1-2 infections
	Moderate (2)	3 infections
	Severe (3)	>3 infections or recurrent infections
Severity of other (viral/bacterial/fungal/parasite) infections (in the last 6 months)	None (0)	No other infections
	Mild (1)	No treatment necessary
	Moderate (2)	Topical treatment necessary
	Severe (3)	Systemic treatment necessary
Other autoimmune/autoinflammatory conditions (that have not been scored yet)		
Current other autoimmune/autoinflammatory manifestation, e.g. AI Thyroiditis (or suspicion of)	None (0)	no
	Mild (1)	1 other autoimmune/autoinflammatory organ manifestation
	Moderate (2)	2 other autoimmune/autoinflammatory organ manifestations

	Severe (3)	3 or more other autoimmune/autoinflammatory organ manifestations

Calculation: The *NFKB1*-DAS is indicated as a percentage value corresponding to the score points achieved relative to the maximum score value. The maximum possible score value results from the maximum number of points of all parameters with available data. For calculation, add all the achieved points and divide that number by the maximum possible score value (= number of parameters with available data times three).

$$NFKB1 - \text{DAS (in \%)} = \frac{\text{sum of achieved score points}}{\text{max. score value (number of parameters with available data x 3)}}$$

Example: A patient with Hypogammaglobulinemia under IVIG substitution (3 points), chronic upper respiratory tract symptoms (2 points) and a VZV reactivation (1 point) with systemic antiviral treatment in the last 6 months (3 points) would achieve 9 points. Because there was no ultrasound data available, the parameter “liver involvement” and “lymphadenopathy” can not be evaluated. This results in a maximum possible score value of 57 points (63 – 6), leading to a *NFKB1*-DAS of 16% (9 / 57).

References

1. Fliegauf M, Kinnunen M, Posadas-Cantera S, Camacho-Ordonez N, Abolhassani H, Alsina L, et al. Detrimental NFKB1 missense variants affecting the Rel-homology domain of p105/p50. Front Immunol. 2022;13:965326. doi:10.3389/fimmu.2022.965326 PubMed PMID: 36105815; PubMed Central PMCID: PMC9465457.

2. Li J, Lei WT, Zhang P, Rapaport F, Seeleuthner Y, Lyu B, et al. Biochemically deleterious human NFKB1 variants underlie an autosomal dominant form of common variable immunodeficiency. J Exp Med. 2021 Sep 2;218(11):e20210566. doi:10.1084/jem.20210566 PubMed PMID: 34473196; PubMed Central PMCID: PMC8421261.

3. Fliegauf M, Nitschke K, Mrovecova P, Posadas-Cantera S, Camacho-Ordonez N, Warnatz K, et al. Compound heterozygous NFKB1 missense variants in cis associated with immunodeficiency. Clin Immunol. 2026 Jan;282:110637. doi:10.1016/j.clim.2025.110637 PubMed PMID: 41202999.

4. Fliegauf M, Krüger R, Steiner S, Hanitsch LG, Büchel S, Wahn V, et al. A Pathogenic Missense Variant in NFKB1 Causes Common Variable Immunodeficiency Due to Detrimental Protein Damage. Front Immunol. 2021;12:621503. doi:10.3389/fimmu.2021.621503 PubMed PMID: 33995346; PubMed Central PMCID: PMC8115018.

5. Agarwal S, Cunningham-Rundles C. Treatment of hypogammaglobulinemia in adults: a scoring system to guide decisions on immunoglobulin replacement. J Allergy Clin Immunol. 2013 Jun;131(6):1699–701. doi:10.1016/j.jaci.2013.01.036 PubMed PMID: 23518142; PubMed Central PMCID: PMC4102312.

6. 207 IgG-Subklassen- und IgA - Mangelsyndrome [Internet]. [cited 2024 Aug 30]. Available from: https://www.imd-berlin.de/fachinformationen/diagnostikinformationen/igg-subklassen-und-iga-mangelsyndrome

7. Krausz M, Uhlmann A, Rump IC, Ihorst G, Goldacker S, Sogkas G, et al. The ABACHAI clinical trial protocol: Safety and efficacy of abatacept (s.c.) in patients with CTLA-4 insufficiency or LRBA deficiency: A non controlled phase 2 clinical trial. Contemp Clin Trials Commun. 2022 Sep 24;30:101008. doi:10.1016/j.conctc.2022.101008 PubMed PMID: 36262801; PubMed Central PMCID: PMC9573884.

8. Neutropenie - Hämatologie und Onkologie - MSD Manual Profi-Ausgabe [Internet]. [cited 2024 Aug 30]. Available from: https://www.msdmanuals.com/de-de/profi/h%C3%A4matologie-und-onkologie/leukopenien/neutropenie

9. Freeman AM, Matto P. Adenopathy. In: StatPearls [Internet]. Treasure Island (FL): StatPearls Publishing; 2023 [cited 2023 Mar 23]. Available from: http://www.ncbi.nlm.nih.gov/books/NBK513250/ PubMed PMID: 30020622.

10. Duale Reihe Innere Medizin - eRef, Thieme [Internet]. [cited 2024 Aug 30]. Available from: https://eref-thieme-de.ezproxy-unifr-1.redi-bw.de/ebooks/2263057

11. Medical Research Council (MRC) [Internet]. 2024 [cited 2024 Aug 30]. Available from: https://www.ukri.org/councils/mrc/

12. MRC Dyspnoea Scale | Primary Care Respiratory Society [Internet]. [cited 2024 Aug 30]. Available from: https://www.pcrs-uk.org/mrc-dyspnoea-scale

13. Wells G, Becker JC, Teng J, Dougados M, Schiff M, Smolen J, et al. Validation of the 28-joint Disease Activity Score (DAS28) and European League Against Rheumatism response criteria based on C-reactive protein against disease progression in patients with rheumatoid arthritis, and comparison with the DAS28 based on erythrocyte sedimentation rate. Annals of the Rheumatic Diseases. 2009 Jun 1;68(6):954–60. doi:10.1136/ard.2007.084459

14. Disease Activity Score-28 for Rheumatoid Arthritis with CRP (DAS28-CRP) [Internet]. [cited 2024 Aug 30]. Available from: https://www.mdcalc.com/calc/2193/disease-activity-score-28-rheumatoid-arthritis-crp-das28-crp

15. J SS, N R, D P, K B, S S, B Z, et al. Severe COVID-19 Is Marked by a Dysregulated Myeloid Cell Compartment. Cell. 2020 Sep 17;182(6). doi:10.1016/j.cell.2020.08.001 PubMed PMID: 32810438.

16. Schuyler RP, Jackson C, Garcia-Perez JE, Baxter RM, Ogolla S, Rochford R, et al. Minimizing Batch Effects in Mass Cytometry Data. Front Immunol. 2019;10:2367. doi:10.3389/fimmu.2019.02367 PubMed PMID: 31681275; PubMed Central PMCID: PMC6803429.
